# On the modeling of the interaction between tumor growth and the immune system using some new fractional and fractional-fractal operators

**DOI:** 10.1186/s13662-020-03040-x

**Published:** 2020-10-19

**Authors:** Behzad Ghanbari

**Affiliations:** 1 Department of Engineering Science, Kermanshah University of Technology, Kermanshah, Iran; 2 Department of Mathematics, Faculty of Engineering and Natural Sciences, Bahçeşehir University, 34349 Istanbul, Turkey

**Keywords:** Mathematical modeling, Fractional and fractional-fractal derivatives, Tumor growth modeling, The immune system, Numerical simulations, Sensitivity analysis

## Abstract

Humans are always exposed to the threat of infectious diseases. It has been proven that there is a direct link between the strength or weakness of the immune system and the spread of infectious diseases such as tuberculosis, hepatitis, AIDS, and Covid-19 as soon as the immune system has no the power to fight infections and infectious diseases. Moreover, it has been proven that mathematical modeling is a great tool to accurately describe complex biological phenomena. In the recent literature, we can easily find that these effective tools provide important contributions to our understanding and analysis of such problems such as tumor growth. This is indeed one of the main reasons for the need to study computational models of how the immune system interacts with other factors involved. To this end, in this paper, we present some new approximate solutions to a computational formulation that models the interaction between tumor growth and the immune system with several fractional and fractal operators. The operators used in this model are the Liouville–Caputo, Caputo–Fabrizio, and Atangana–Baleanu–Caputo in both fractional and fractal-fractional senses. The existence and uniqueness of the solution in each of these cases is also verified. To complete our analysis, we include numerous numerical simulations to show the behavior of tumors. These diagrams help us explain mathematical results and better describe related biological concepts. In many cases the approximate results obtained have a chaotic structure, which justifies the complexity of unpredictable and uncontrollable behavior of cancerous tumors. As a result, the newly implemented operators certainly open new research windows in further computational models arising in the modeling of different diseases. It is confirmed that similar problems in the field can be also be modeled by the approaches employed in this paper.

## Introduction

The immune system is a real masterpiece that does extraordinary things every day while we do not realize such numerous activities. The job of the immune system is protecting our bodies against invading factors such as bacteria and viruses. Many factors such as low nutrient intake, lack of sleep, and high stress weaken a person’s immune system. On the other hand, people with cancer are also at risk for infectious diseases since they usually undergo special chemotherapy treatments using special drugs that weaken their immune system. The main purpose of these special drugs is destroying diseased cells without damaging adjacent tissues. Unfortunately, some healthy cells and tissues in the body may also be affected during this treatment.

Nowadays, with the pandemic outbreak of the dangerous infectious disease COVID-19 all over the world, infectious disease specialists and epidemiologists constantly emphasize the strengthening of the immune system as one of the most important ways to prevent this deadly disease.

In this paper, we provide some novel approximate solutions to a computational model that formulates the interaction between tumor growth and the immune system, including several fractional and fractal operators. The model consists of three state variables, each representing the number of specific cells in the problem. The interaction between these variables is presented by Itik and Banks [26] through following nonlinear differential equation system:

1 $$ \begin{gathered} \frac{d\mathsf{T}(\tau )}{d\tau }=k'_{1} \mathsf{T} (\tau ) \biggl(1- \frac{\mathsf{T}(\tau )}{s_{1}} \biggr)-\beta '_{12} \mathsf{T}(\tau ) \mathsf{H}(\tau )-\beta '_{13} \mathsf{T}(\tau )\mathsf{E}(\tau ), \\ \frac{d\mathsf{H}(\tau )}{d\tau }=k'_{2} \mathsf{H} (\tau ) \biggl(1- \frac{\mathsf{H}(\tau )}{s_{2}} \biggr)-\beta _{21}' \mathsf{T}(\tau ) \mathsf{H}(\tau ), \\ \frac{d\mathsf{E}(\tau )}{d\tau }= \frac{k_{3} \mathsf{T}(\tau )\mathsf{E}(\tau )}{\mathsf{T}(\tau )+s_{3}}- \beta _{31}' \mathsf{T}(\tau )\mathsf{E}(\tau )-c'_{3} \mathsf{E}( \tau ), \end{gathered} $$

subject to initial conditions $(\mathsf{T}(0),\mathsf{H}(0),\mathsf{E}(0) )= ( \mathsf{T}_{0},\mathsf{H}_{0},\mathsf{E}_{0} )\geq 0$.

In this model, $\mathsf{T}(\tau )$ is used to count the number of tumor cells at time *τ*, $\mathsf{H}(\tau )$ represents the number of healthy host cells, and $\mathsf{E}(\tau )$ refers to the number of effector immune cells in the single tumor-site compartment. Moreover, *τ* represents the rate of change in the population of the tumor cells, $k_{1}'$ describes the growth of the tumor cells, and $s_{1}'$ is their maximum carrying capacity. The first term of Eq. (1) expresses to the logistic growth of the tumor cells in the absence of any effect from other cell populations. The competition between the host cells $\mathsf{H}(\tau )$ and the tumor cells $\mathsf{T}(\tau )$, which results in the loss of the tumor cell population, is given by the term $\beta '_{12}\mathsf{T(\tau )H(\tau )}$. Moreover, $\beta '_{12}$ refers to the tumor cell killing rate by the effector cells $\mathsf{E}(\tau )$. In the second equation of system (1) the healthy tissue cells also grow logistically with the growth rate $k'_{2}$ and maximum carrying capacity $s'_{2}$. We assume that the cancer cells proliferate faster than the healthy cells, and thus $k_{1}' > k_{2}'$. The tumor cells also inactivate the healthy cells at the rate $\beta '_{21}$. Also, the third equation of system (1) illustrates the stimulation of the immune system by the tumor cells with tumor specific antigens. One of the other assumptions considered in the model is that the immune system depends directly on the number of tumor cells a the rate of positive constants $k'_{3}$ and $s'_{3}$. Finally, $\beta '_{31}$ is the rate of change corresponding to the inactivated effector cells by the tumor cells, and $c_{3}'$ is their rate of natural death. All parameters in the model are positive constants.

For simplicity of analysis, we first nondimensionalize system (1) by employing the definitions

2 $$ \mathcal{T}(t)=\frac{\mathsf{T}(\tau )}{s_{1}}, \qquad \mathcal{H}(t)= \frac{\mathsf{H}(\tau )}{s_{2}}, \qquad \mathcal{E}(t)= \frac{\mathsf{E}(\tau )}{s_{3}}, \quad t= {k_{1}} { \tau }. $$

In addition, we use the following new parameters:

3 $$ \begin{gathered} \beta _{31}= \frac{\beta _{31}' s_{1}}{ k_{1}} ,\qquad \beta _{13}= \frac{\beta _{13}' s_{3}}{ k_{1}} ,\qquad \beta _{12}= \frac{\beta _{12}' s_{2}}{ k_{1}} , \qquad \beta _{31}= \frac{\beta _{31}' s_{1}}{ k_{1}},\\ k_{2}=\frac{k_{2}'}{k_{1}},\qquad k_{3}= \frac{k_{3}'}{k_{1}},\qquad s_{3}=\frac{s_{3}'}{s1}, \qquad c_{3}= \frac{c_{3}'}{k_{1}}. \end{gathered} $$

By involving the introduced changes in equations (2) and (3), we obtain a new dimensionless form for the problem [26]:

4 $$ \begin{gathered} \frac{d \mathcal{T}(t)}{dt}= \mathcal{T} (t) \bigl(1-\mathcal{T}(t) \bigr)-\beta _{12} \mathcal{T}(t)\mathcal{H}(t)- \beta _{13} \mathcal{T}(t)\mathcal{E}(t), \\ \frac{d \mathcal{H}(t)}{dt}=k_{2} \mathcal{H} (t) \bigl(1-\mathcal{H}(t) \bigr)-\beta _{21} \mathcal{T}(t)\mathcal{H}(t), \\ \frac{d \mathcal{E}(t)}{dt}= \frac{k_{3} \mathcal{T}(t)\mathcal{E}(t)}{\mathcal{T}(t)+s_{3}}- \beta _{31} \mathcal{T}(t) \mathcal{E}(t)-c_{3} \mathcal{E}(t). \end{gathered} $$

Due to the great importance of model (4), many researchers have shown interest in examining various technical and computational aspects of this model. The authors explain the biological relevance of model (7). In [41] the authors have utilized the localization of compact invariant sets (LCIS) method and Lyapunov stability theory to investigate the global dynamics of model (4). In [28] the authors have focused on the mathematical points of view, such as the existence of Hopf bifurcations corresponding to the model. In [24] the authors considered the model using the Caputo–Fabrizio–Caputo and new fractional derivative with Mittag-Leffler kernel in the Liouville–Caputo sense. Starko and Coria [40] provided sufficient conditions on model parameters and treatment parameters under which all trajectories in the positive orthant tend to the tumor-free equilibrium point. The authors in [32] have developed an efficient methodology of partial control and applied it to avoid the extinction of the healthy tissue. In [42], taking impulsive differential equations into account, the authors have presented a mathematical formulation of tumor–immune interaction with periodically pulsed immunotherapy. In [3], model (4) was modified to include three delay parameters in the problem.

Fractional calculus has a relatively long history almost as long as a integer-order differential account. However, in recent decades, the implementations of these concepts was neglected compared to standard calculus. This trend seems to have changed in the past years in general. A defining sign of this change is the increasing use of these tools in the literature. Thanks to the researchers’ efforts, many differentiated and integral operators based on different approaches were proposed and then successfully implemented in the past few years [1, 4, 5, 10, 13, 23, 25, 30]. From the perspective of numerical aspects, a wide range of new mathematical methods was successfully applied in various branches of science [6, 13, 16–19, 27, 29, 35, 37, 39]. For example, in [9] the authors have developed an efficient numerical treatment for ordinary fractional and fractal-fractional differential differential equations, which is based on Newton polynomials. In [8], Atangana and his collaborator have successfully applied a new numerical algorithm to approximate a modified version of the Chua attractor model with both fractional and fractal-fractional operators. An efficient numerical technique, the Atangana–Seda numerical scheme, based on Newton polynomials, is utilized in [7] to handle a chaotic problem with fractional operators, which include the exponential decay, power law, and Mittag-Leffler kernel. In [23] the author has employed some novel differential and integral operators of fractional order and fractal dimension using he uville–Caputo and Atangana–Baleanu definitions to obtain multiple attractors and periodicity on the Vallis model for El Niño/La Niña-Southern oscillation model. A new fractional-order compartmental SEIRS model with Caputo-type fractional-order derivative was also studied in [25]. Chaotic systems are almost one of the most important and applicable types of nonlinear equations [12, 21, 34, 36, 38]. Therefore in many cases the exact solution is not available for such equations. On the other hand, the use of new derivative operators in structures of chaotic systems has made significant development in this field [2, 22]. In some cases the researchers have obtained desirable attractors, which were not achievable by common integer-order operators. This fact highlights the importance of new derivative operators in other real-world models. Motivated by these achievements, especially following the work [24], we intend to investigate the model presented in equation (4) using some new efficient fractional and fractional-fractional operators.

To reach this goal, the subsequent parts of the paper are structured as follows. The analysis of model equilibrium points is presented in Sect. 2. This model is examined in the next section via the Liouville–Caputo fractional-order derivative. This section also confirms that under appropriate assumptions, the model always possesses a unique solution. Then a numerical method corresponding to this structure is designed and then utilized. Besides, detailed numerical simulations are presented. Similar processes will be followed in Sects. 4 and 5 of the paper, with the Caputo–Fabrizio–Caputo and Atangana–Baleanu–Caputo fractional derivative operators, respectively. In Sect. 6, we examine the model via several fractal-fractional operators. This section also presents the numerical methods corresponding to each of these operators. To investigate the dynamic behavior of the results, we added several numerical simulations. Finally, in Sect. 7, we present a summary of the results and achievements of the paper.

## Investigation of stability of equilibrium points of models

In this section, we analyze the equilibrium points of the considered model (4). These points are in fact the roots of the nonlinear algebraic system that is contracted on the right-hand side of the model. By solving this system, we determine six possible equilibrium points for the model [28].

*Point 1:* the no “living cell” singular point $\mathcal{P}_{1}=(0,0,0)$.

*Point 2:* The tumor-free fixed point $\mathcal{P}_{2}=(0,1,0)$.

*Point 3:* The fixed point $\mathcal{P}_{3}=( 1,0,0)$, which implies the existence of tumor cells in the model.

*Point 4:* The fixed point $\mathcal{P}_{4}=( {\mathcal{T}^{*}},0, \frac{1-{\mathcal{T}^{*}}}{\beta _{13}})$. The first coordinate ${\mathcal{T}^{*}}$ is determined by finding the nonnegative root(s) of the characteristic equation

5 $$ \beta _{13}{\mathcal{T}^{*}}^{2}+{ \mathcal{T}^{*}} (c_{3}+s_{3} \beta _{31}-k_{3} )+s_{3} c_{3}=0. $$

This equation has the acceptable root

$$ {\mathcal{T}^{*}}= \frac{k_{3}-c_{3}-s_{3}\beta _{31}+\sqrt{ (k_{3}-c_{3}-s_{3}\beta _{31} )^{2}-4s_{3} c_{3} \beta _{31}}}{2\beta _{31}}. $$

Necessary conditions for the existence of this equilibrium point are

$$ k_{3}>c_{3}+s_{3} \beta _{31}, \qquad {\mathcal{T}^{*}}< 1. $$

*Point 5:* The singular point $\mathcal{P}_{5}=( { \frac{{ k2} ( \beta _{12}-1 ) }{\beta 12 \beta 21-{ k2}}}, { \frac{\beta 21-{ k2}}{\beta _{12} \beta _{21}-{ k2}}}, 0 )$, which implies the coexistence of cancer and host cells. The necessary conditions for the existence of this equilibrium point are

$$ \beta _{21}>{ k_{2}}, \qquad \beta _{12}>1, \qquad \beta _{12} \beta _{21}> k_{2}. $$

It should also be noted that for $\beta _{12}=1$, this equilibrium point becomes the equilibrium point $\mathcal{P}_{2}$.

*Point 6:* The interior fixed point $\mathcal{P}_{6}=( {\mathcal{T}^{*}}, \frac{k_{2}-\beta _{21}{\mathcal{T}^{*}}}{k_{2}}, \frac{k_{2}(1-\beta _{12})+{\mathcal{T}^{*}} (\beta _{21}\beta _{12}-k_{2} )}{k_{2}\beta _{13}})$, where ${\mathcal{T}^{*}}$ is a positive root of $\beta _{13}{\mathcal{T}^{*}}^{2}+{\mathcal{T}^{*}} (c_{3}+s_{3} \beta _{31}-k_{3} )+s_{3} c_{3}=0$, as mentioned earlier. In this case, all three cell populations are present in the problem. The necessary conditions for the existence of this equilibrium point are

$$ { k_{3}}>c_{3}+s_{3}\beta _{31}, \qquad \beta _{12}< 1,\qquad \beta _{12} \beta _{21}>k_{2}, \qquad \beta _{21}> k_{2}, \qquad {\mathcal{T}^{*}}< \frac{k_{2}}{\beta _{21}}. $$

Moreover, the Jacobi matrix corresponding to this system is

6 $$ \begin{aligned} &J\bigl({\mathcal{T}^{*}},{ \mathcal{H}^{*}},{\mathcal{E}^{*}}\bigr) \\ &\quad = \begin{bmatrix} -\beta _{12}{\mathcal{H}^{*}}-\beta _{13}{\mathcal{E}^{*}}-2{ \mathcal{T}^{*}}+1&-\beta _{12}{\mathcal{T}^{*}}& -\beta _{13}{ \mathcal{T}^{*}} \\ -{\mathcal{H}^{*}}\beta _{21}&{ k_{2}} ( 1-{\mathcal{H}^{*}} ) -{ k_{2}}{\mathcal{H}^{*}}-\beta 21{\mathcal{T}^{*}}&0 \\ {\frac{{ k_{3} }{\mathcal{E}^{*}}}{{\mathcal{T}^{*}}+{ s_{3}}}}-{ \frac{{ k_{3}}{\mathcal{T}^{*}}{\mathcal{E}^{*}}}{ ( {\mathcal{T}^{*}}+{ s_{3}} ) ^{ 2}}}-\beta _{31}{\mathcal{E}^{*}}&0&{ \frac{{ k_{3}}{\mathcal{T}^{*}}}{{\mathcal{T}^{*}}+{ s_{3}}}}-\beta _{31}{ \mathcal{T}^{*}}-{ c_{3} }\end{bmatrix} .\end{aligned} $$

## The model via the Liouville–Caputo fractional derivative

In this section, we consider model (4) with the Liouville–Caputo (LC) fractional derivative,

7 $$ \begin{gathered} {}_{0}^{\mathsf{LC}} \mathcal{D}_{t}^{{\boldsymbol{\alpha }}} {\mathcal{T}}(t)= \mathcal{T} (t) \bigl(1- \mathcal{T}(t) \bigr)-\beta _{12} \mathcal{T}(t)\mathcal{H}(t)-\beta _{13} \mathcal{T}(t)\mathcal{E}(t), \\ {}_{0}^{\mathsf{LC}} \mathcal{D}_{t}^{{\boldsymbol{\alpha }}} { \mathcal{H}}(t)=k_{2} \mathcal{H} (t) \bigl(1-\mathcal{H}(t) \bigr)- \beta _{21} \mathcal{T}(t)\mathcal{H}(t), \\ {}_{0}^{\mathsf{LC}} \mathcal{D}_{t}^{{\boldsymbol{\alpha }}} { \mathcal{E}}(t)= \frac{k_{3} \mathcal{T}(t)\mathcal{E}(t)}{\mathcal{T}(t)+s_{3}}- \beta _{31} \mathcal{T}(t) \mathcal{E}(t)-c_{3} \mathcal{E}(t), \end{gathered} $$

where the LC fractional derivative is defined as [14]

8 $$ {}_{0}^{\mathsf{\mathsf{LC}}} \mathcal{D}_{t}^{{\boldsymbol{\alpha }}} { \mathcal{T}}(t)=\frac{1}{\Gamma {(\alpha })} \int _{0}^{t} (t-{\eta })^{ \alpha -1}\phi '({\eta })\, d{\eta },\quad 0< {\boldsymbol{\alpha }}\le 1. $$

Utilizing the Laplace transform on the LC derivative (8), we get

9 $$ \mathcal{L} \bigl\{ {{}^{\mathsf{LC}}_{0}} {\mathcal{D}}_{t}^{{\boldsymbol{\alpha }}} \phi (t) \bigr\} = s^{{\boldsymbol{\alpha }}} \mathcal{L} \bigl\{ \phi (t) \bigr\} - \sum _{k = 0}^{m-1}s^{{\boldsymbol{\alpha }} - k - 1} \phi ^{(k)}(0),\quad m= \lceil {\boldsymbol{\alpha }}\rceil . $$

Taking (9) into account and then utilizing the inverse Laplace transform on Eq. (7), we get

10 $$ \begin{gathered} {\mathcal{T}}(t)={\mathcal{T}}(0)+ \mathcal{L}^{-1} \biggl\{ \frac{1}{s^{{\boldsymbol{\alpha }}}}\mathcal{L} \bigl[ \mathcal{T} (t) \bigl(1- \mathcal{T}(t) \bigr)-\beta _{12} \mathcal{T}(t)\mathcal{H}(t)- \beta _{13} \mathcal{T}(t)\mathcal{E}(t) \bigr] \biggr\} , \\ {\mathcal{H}}(t)={\mathcal{H}}(0)+\mathcal{L}^{-1} \biggl\{ \frac{1}{s^{{\boldsymbol{\alpha }}}}\mathcal{L} \bigl[k_{2} \mathcal{H} (t) \bigl(1- \mathcal{H}(t) \bigr)-\beta _{21} \mathcal{T}(t)\mathcal{H}(t) \bigr] \biggr\} , \\ {\mathcal{E}}(t)={\mathcal{E}}(0)+\mathcal{L}^{-1} \biggl\{ \frac{1}{s^{{\boldsymbol{\alpha }}}}\mathcal{L} \biggl[ \frac{k_{3} \mathcal{T}(t)\mathcal{E}(t)}{\mathcal{T}(t)+s_{3}}- \beta _{31} \mathcal{T}(t)\mathcal{E}(t)-c_{3} \mathcal{E}(t) \biggr] \biggr\} . \end{gathered} $$

From (10) we suggest the following iterative schemes:

11 $$ \begin{gathered} {\mathcal{T}_{n}}(t)={ \mathcal{T}_{0}}+\mathcal{L}^{-1} \biggl\{ \frac{1}{s^{{\boldsymbol{\alpha }}}} \mathcal{L} \bigl[\mathcal{T}_{n-1} (t) \bigl(1-\mathcal{T}_{n-1}(t) \bigr)-\beta _{12} \mathcal{T}_{n-1}(t) \mathcal{H}_{n-1}(t)- \beta _{13} x_{n-1}(t)\mathcal{E}_{n-1}(t) \bigr] \biggr\} , \\ {\mathcal{H}_{n}}(t)={\mathcal{H}_{0}}+ \mathcal{L}^{-1} \biggl\{ \frac{1}{s^{{\boldsymbol{\alpha }}}}\mathcal{L} \bigl[k_{2} \mathcal{H}_{n-1} (t) \bigl(1-\mathcal{H}_{n-1}(t) \bigr)-\beta _{21} \mathcal{T}_{n-1}(t)y_{n-1}(t) \bigr] \biggr\} , \\ {\mathcal{E}_{n}}(t)={\mathcal{E}_{0}}+ \mathcal{L}^{-1} \biggl\{ \frac{1}{s^{{\boldsymbol{\alpha }}}}\mathcal{L} \biggl[ \frac{k_{3} \mathcal{T}_{n-1}(t)\mathcal{E}_{n-1}(t)}{\mathcal{T}_{n-1}(t)+s_{3}}- \beta _{31} \mathcal{T}_{n-1}(t) \mathcal{E}_{n-1}(t)-c_{3} \mathcal{E}(t) \biggr] \biggr\} , \end{gathered} $$

where

12 $$ \mathcal{T}_{0}={\mathcal{T}}(0),\qquad \mathcal{H}_{0}={ \mathcal{H}}(0), \qquad \mathcal{E}_{0}={\mathcal{E}}(0). $$

The desired approximate solutions can be obtained by computing the limits

13 $$ \mathcal{T}(t)=\lim_{n\rightarrow \infty } {\mathcal{T}}_{n}(t), \qquad \mathcal{H}(t)=\lim_{n\rightarrow \infty } {\mathcal{H}}_{n}(t), \qquad \mathcal{E}(t)=\lim_{n\rightarrow \infty } {\mathcal{E}}_{n}(t). $$

### Existence and uniqueness

Let us assume that a Banach space like Ω has a closed convex bounded subset Ξ that contains a fixed point of Ω. Moreover, let $\omega : \Xi \rightarrow \Xi $ be a condensing map. Moreover, let us assume that there exists $\Delta \in (0,\xi )$ such that . The exist functions  such that , , and . Then we have:

, , and  are Lipschitz and bounded.
, , and  are compact and bounded.
3. $|R(t,m)-R(t,z)|\leq L_{1}(t)\|m-z\|$.

With the help of the Riemann–Liouville integral, Eq. (7) is written as 14

#### Theorem 1

*As soon as*

15 $$ \psi =\frac{\upsilon \Vert L \Vert _{1/\triangledown }T^{M}}{\Gamma (\rho )}< 1, $$

*then under two properties* 1 *and* 2, *the existence of a solution to the problem is guaranteed*.

#### Proof

Let us take *χ* such that $\tau (0)+\frac{1}{\Gamma (\rho )}\upsilon (\|H_{1}\|_{1/ \triangledown }+\|H_{2}\|_{1/\triangledown })T^{M}\leq \chi $, and let $\Xi _{\mu }=\{m:\|m\|\leq \chi \}$ be a closed set in the Banach space $([0,T],\Omega )$ equipped with the norm of $\sup \|\cdot \|$.

Then, by the definition $m:\Xi _{\chi }\rightarrow ([0,T],\Omega )$, , we have 16 Hence we obtain that , , , and  are condensing, and the fixed points of , , , and  are verified.

I) Let us show that . For $m \in \Xi _{\chi }$, we have 17 Similarly, we have  and 18 and therefore .

II) Let us show that , , and  are contractions. For $m,z \in \Xi _{\chi }$, we conclude  where

19 $$ \Psi _{i}= \frac{ \sigma _{i} \Vert L \Vert _{1/\triangledown }T^{\mu _{i}}}{\Gamma (\rho )}< 1, \quad 1\leq i \leq 3. $$

These statements confirm that , , and  are contractions.

III) Let us show that , , and  are compact. For $0\leq j_{1} \leq j_{2} \leq T$, we have 20 Similarly, we get 21 Finally, we have  for $\sigma _{i}, 1\leq i \leq 3$.

Now by the Arzelà–Ascoli theorem [15] , , and  are relatively compact. So , , and  are compact.

Since , , , and  are contractions and ,  and  are compact and therefore continuous, the maps , , and  are condensing on $\Xi _{\xi }$. Hence the existence of a fixed point for each point , , and  is proved.

IV) We will show that the problem has a unique solution. To this end, let us define the map *H* as follows: 22

For , , , , , , we obtain 23 Besides, we have  and  These results indicate that model (7) will always has a unique solution. □

### The numerical method

In this section, we use the Adams–Bashforth–Moulton (ABM) numerical method. Here we follow the steps to apply this method in solving the following fractional-order problem:

24 $$ {}^{\mathsf{C}}_{0}{\mathcal{D}}_{t}^{{\boldsymbol{\alpha }}}\phi (t)=\Xi \bigl(t,\phi (t)\bigr), \quad \phi ^{k}(0)=\phi ^{k}_{0}, k=0,1,\ldots,n-1. $$

Now taking the Liouville–Caputo fractional integration on (24) yields

25 $$ \phi (t)=\sum_{k=0}^{n-1}f_{0}^{(k)} \frac{t^{k}}{k!}+ \frac{1}{\Gamma ({\boldsymbol{\alpha }})} \int _{0}^{t}(t-{\eta })^{{ \boldsymbol{\alpha }}-1}\Xi \bigl({ \eta },\phi ({\eta })\bigr)\, d{\eta }. $$

The following predictor–corrector form determines an approximate solution to the problem [31]:

26 $$ \begin{gathered} \phi ^{P}_{k+1}= \sum_{\zeta =0}^{n-1} \frac{t^{\zeta }_{k+1}}{\zeta !}\phi ^{(\zeta )}_{0}+ \frac{1}{\Gamma ({\boldsymbol{\alpha }})}\sum _{\zeta =0}^{k}\theta _{ \zeta ,k+1}\Xi (t_{\zeta },f_{\zeta }), \\ \phi _{k+1}=\sum_{\zeta =0}^{n-1} \frac{t^{\zeta }_{k+1}}{\zeta !}\phi ^{(\zeta )}_{0}+ \frac{1}{\Gamma ({\boldsymbol{\alpha }})} \Biggl( \sum_{\zeta =0}^{k} \gamma _{\zeta ,k+1}\Xi (t_{\zeta },f_{\zeta })+\gamma _{k+1,k+1}\Xi \bigl(t_{k+1}, \phi ^{P}_{k+1}\bigr) \Biggr), \end{gathered} $$

where

27 $$\begin{aligned}& \gamma _{\zeta ,k+1}= \frac{\hbar ^{{\boldsymbol{\alpha }}}}{{\boldsymbol{\alpha }}({\boldsymbol{\alpha }}+1)} \cdot \textstyle\begin{cases} k^{{\boldsymbol{\alpha }}+1}-(k-{\boldsymbol{\alpha }})(k+1)^{{\boldsymbol{\alpha }}}, &\zeta =0, \\ (-\zeta +k+2)^{{\boldsymbol{\alpha }}+1}+(-\zeta +k)^{{\boldsymbol{\alpha }}+1}-2(- \zeta +k+1)^{{\boldsymbol{\alpha }}+1}, &1\leq \zeta \leq k, \\ 1, &\zeta =k+1, \end{cases}\displaystyle \\& \theta _{\zeta ,k+1}=\frac{\hbar ^{{\boldsymbol{\alpha }}}}{{\boldsymbol{\alpha }}}\bigl((- \zeta +k+1)^{{\boldsymbol{\alpha }}}-(- \zeta +k)^{{\boldsymbol{\alpha }}}\bigr), \quad \zeta =0,1,2,\ldots,k. \end{aligned}$$

Utilizing the numerical algorithm presented in (26), we determine an approximate solution to the fractional problem (7) from the formulae

28 $$ \begin{gathered} \mathcal{T}(t)=\sum_{k=0}^{n-1} \mathcal{T}^{(k)}(0) \frac{t^{k}}{k!}\\ \hphantom{\mathcal{T}(t)=}{}+\frac{1}{\Gamma ({\boldsymbol{\alpha }})} \int _{0}^{t}(t-{ \eta })^{{\boldsymbol{\alpha }}-1} \bigl[ \mathcal{T} ({\eta }) \bigl(1- \mathcal{T}({\eta }) \bigr)-\beta _{12} \mathcal{T}({\eta }) \mathcal{H}({\eta })-\beta _{13} \mathcal{T}({\eta })\mathcal{E}({\eta }) \bigr]d{\eta }, \\ \mathcal{H}(t)=\sum_{k=0}^{n-1} \mathcal{H}^{(k)}(0) \frac{t^{k}}{k!}+\frac{1}{\Gamma ({\boldsymbol{\alpha }})} \int _{0}^{t}(t-{ \eta })^{{\boldsymbol{\alpha }}-1} \bigl[ k_{2} \mathcal{H} ({\eta }) \bigl(1- \mathcal{H}({\eta }) \bigr)-\beta _{21} \mathcal{T}({\eta }) \mathcal{H}({\eta }) \bigr]d{\eta }, \\ \mathcal{E}(t)=\sum_{k=0}^{n-1} \mathcal{E}^{(k)}(0) \frac{t^{k}}{k!}+\frac{1}{\Gamma ({\boldsymbol{\alpha }})} \int _{0}^{t}(t-{ \eta })^{{\boldsymbol{\alpha }}-1} \biggl[ \frac{k_{3} \mathcal{T}({\eta })\mathcal{E}({\eta })}{\mathcal{T}({\eta })+s_{3}}- \beta _{31} \mathcal{T}({\eta })\mathcal{E}({\eta })-c_{3} \mathcal{E}({ \eta }) \biggr]d{\eta }. \end{gathered} $$

### Numerical simulations

**Figure 1:**
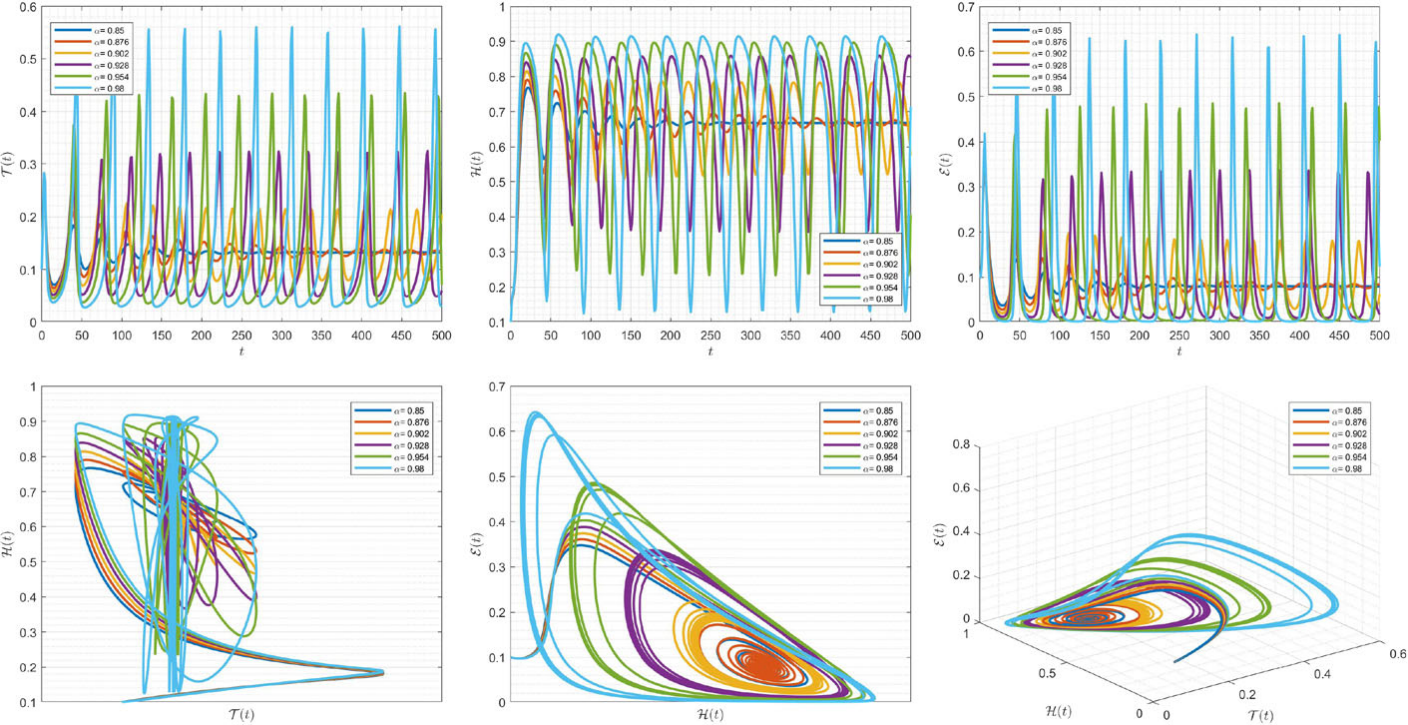
Simulations for solving (7) using (26) along with $(\mathcal{T}(0),\mathcal{H}(0),\mathcal{E}(0) )= (0.1,0.1,0.1 )$

**Figure 2:**
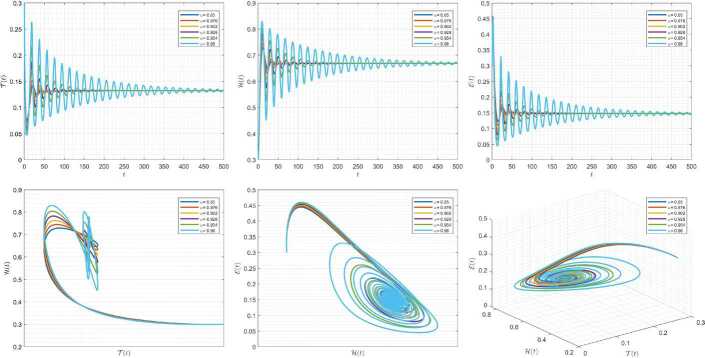
Simulations for solving (7) using (26) along with $(\mathcal{T}(0),\mathcal{H}(0),\mathcal{E}(0) )= (0.3,0.3,0.3 )$ and $\beta _{12}= 0.745$

**Figure 3:**
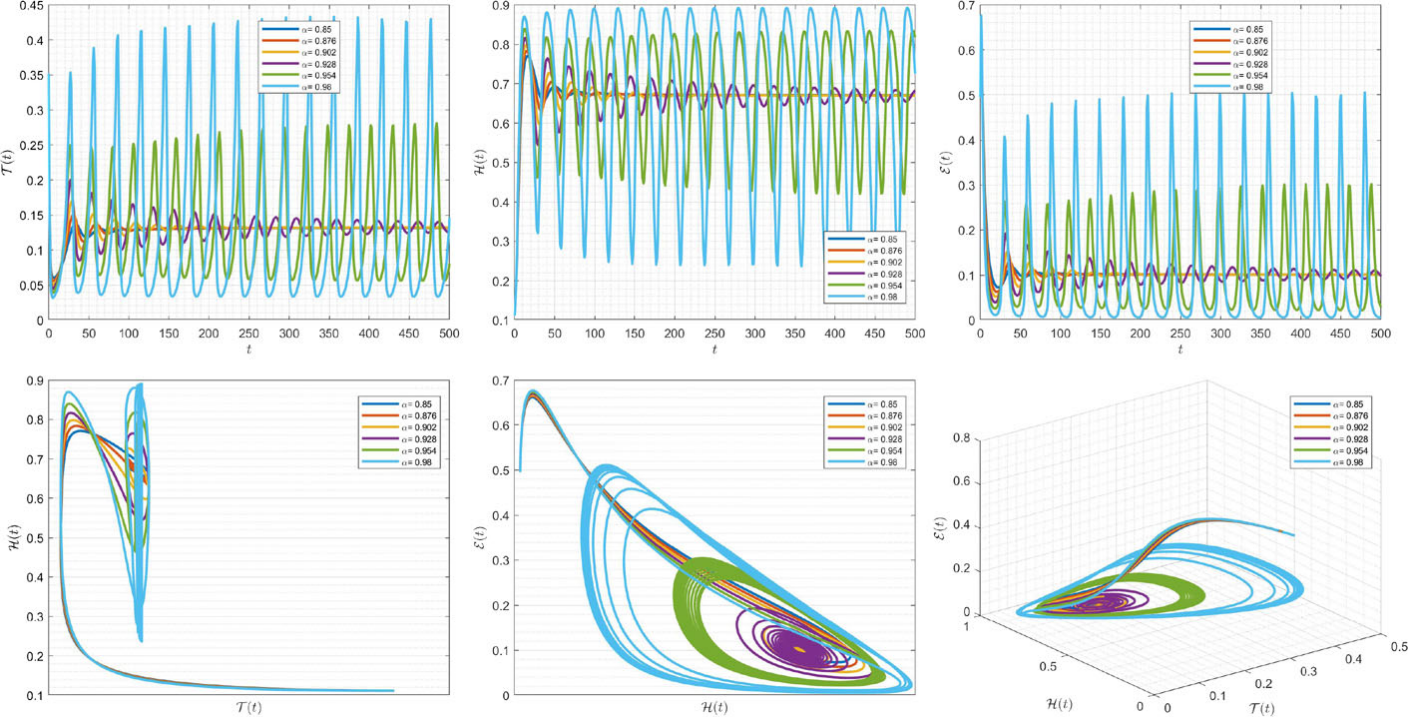
Simulations for solving (7) using (26) along with $(\mathcal{T}(0),\mathcal{H}(0),\mathcal{E}(0) )= ( 0.3517, 0.1115, 0.4951 )$ and $\beta _{12}= 0.92$

Figures 1–3 demonstrate the variation of state variables in model (7) when the scheme (26) is utilized for different values of $\alpha \in (0,1]$. In this simulations, we have considered the following values in the model: $\beta _{12}=1$, $\beta _{13}=2.5$, $k_{2}=0.6$, $\beta _{21}=1.5$, $k_{3}=4.5$, $s_{3}=1$, $a_{31}=0.2$, and $d_{3}=0.5$. In our performed numerical simulations, we take $t_{\mathrm{final}}= 500$ and $\hbar = 0.001$. In Fig. 1, we take $(\mathcal{T}(t), \mathcal{H}(t), \mathcal{E}(t) )|_{t=0}= (0.1, 0.1, 0.1 )$. In this case the model exhibits chaotic attractor behavior. Also, by taking into consideration $(\mathcal{T}(t), \mathcal{H}(t), \mathcal{E}(t) )|_{t=0}= (0.3, 0.3, 0.3 )$, and $\beta _{12} = 0.745 $ the model shows the limit cycle behavior in Fig. 2, whereas for $\mathcal{T}(0)=0.3517$, $\mathcal{H}(0)=0.1115$, $\mathcal{E}(0)=0.4951$, and $\beta _{12} = 0.920$, we get periodic orbit trajectories in the solutions as depicted in Fig. 3.

## The model via Caputo–Fabrizio–Caputo fractional derivative

In this section, we study the following model:

29 $$ \begin{gathered} {}_{0}^{\mathsf{CFC}} \mathcal{D}_{t}^{{\boldsymbol{\alpha }}} {\mathcal{T}}(t)= \mathcal{T} (t) \bigl(1- \mathcal{T}(t) \bigr)-\beta _{12} \mathcal{T}(t)\mathcal{H}(t)-\beta _{13} \mathcal{T}(t)\mathcal{E}(t), \\ {}_{0}^{\mathsf{CFC}} \mathcal{D}_{t}^{{\boldsymbol{\alpha }}} { \mathcal{H}}(t)=k_{2} \mathcal{H} (t) \bigl(1-\mathcal{H}(t) \bigr)- \beta _{21} \mathcal{T}(t)\mathcal{H}(t), \\ {}_{0}^{\mathsf{CFC}} \mathcal{D}_{t}^{{\boldsymbol{\alpha }}} { \mathcal{E}}(t)= \frac{k_{3} \mathcal{T}(t)\mathcal{E}(t)}{\mathcal{T}(t)+s_{3}}- \beta _{31} \mathcal{T}(t) \mathcal{E}(t)-c_{3} \mathcal{E}(t). \end{gathered} $$

The fractional derivative operator ${}_{0}^{\mathsf{CFC}}\mathcal{D}_{t}^{{\boldsymbol{\alpha }}}$ in this model is Caputo–Fabrizio–Caputo (CFC) given by [13]

30 $$ {}_{0}^{\mathsf{\mathsf{CFC}}} \mathcal{D}_{t}^{{\boldsymbol{\alpha }}} {\phi }(t)= \frac{\mathsf{M}({\boldsymbol{\alpha }})}{n-{\boldsymbol{\alpha }}} \int _{0}^{t} \phi '({ \eta })\exp \biggl[-\frac{{\boldsymbol{\alpha }}}{1-{\boldsymbol{\alpha }}}(t-{\eta }) \biggr]\,d{\eta },\quad 0< {\boldsymbol{\alpha }}\le 1, $$

where

31 $$ \mathsf{M}({\boldsymbol{\alpha }})=\frac{2}{2-{\boldsymbol{\alpha }}},\quad 0< { \boldsymbol{\alpha }}< 1. $$

The CFC fractional integral is also defined by [33]

32 $$ {}_{0}^{\mathsf{\mathsf{CFC}}} \mathcal{I}_{t}^{{\boldsymbol{\alpha }}} {\phi }(t)= \frac{2(1-{\boldsymbol{\alpha }}) }{(2-{\boldsymbol{\alpha }})\mathsf{M}({\boldsymbol{\alpha }})} \phi (t)+ \frac{2{\boldsymbol{\alpha }} }{(2-{\boldsymbol{\alpha }})\mathsf{M}({\boldsymbol{\alpha }})} \int _{0}^{t} \phi ({\eta })\,d{\eta },\quad t\ge 0. $$

### Existence of the coupled solutions

Applying the CFC integral definition (29), we obtain the following relationships:

33 $$ \textstyle\begin{cases} {\mathcal{T}}(t)- {\mathcal{T}}(0)= {}_{0}^{\mathsf{\mathsf{CFC}}} \mathcal{I}_{t}^{{\boldsymbol{\alpha }}} \{ \mathcal{T} (t) (1- \mathcal{T}(t) )-\beta _{12} \mathcal{T}(t)\mathcal{H}(t)- \beta _{13} \mathcal{T}(t)\mathcal{E}(t) \}, \\ {\mathcal{H}}(t)- {\mathcal{H}}(0)= {}_{0}^{\mathsf{\mathsf{CFC}}} \mathcal{I}_{t}^{{\boldsymbol{\alpha }}} \{k_{2} \mathcal{H} (t) (1- \mathcal{H}(t) )-\beta _{21} \mathcal{T}(t)\mathcal{H}(t) \}, \\ {\mathcal{E}}(t)- {\mathcal{E}}(0)= {}_{0}^{\mathsf{\mathsf{CFC}}} \mathcal{I}_{t}^{{\boldsymbol{\alpha }}} \{ \frac{k_{3} \mathcal{T}(t)\mathcal{E}(t)}{\mathcal{T}(t)+s_{3}}- \beta _{31} \mathcal{T}(t)\mathcal{E}(t)-c_{3} \mathcal{E}(t) \}. \end{cases} $$

Now we consider the following kernels:

34 $$ \textstyle\begin{cases} \mathcal{K}_{1} (t,{\mathcal{T}}(t),{\mathcal{H}}(t),{ \mathcal{E}}(t) )= \mathcal{T} (t) (1-\mathcal{T}(t) )-\beta _{12} \mathcal{T}(t)\mathcal{H}(t)-\beta _{13} \mathcal{T}(t)\mathcal{E}(t), \\ \mathcal{K}_{2} (t,{\mathcal{T}}(t),{\mathcal{H}}(t),{ \mathcal{E}}(t) )=k_{2} \mathcal{H} (t) (1-\mathcal{H}(t) )-\beta _{21} \mathcal{T}(t)\mathcal{H}(t) , \\ \mathcal{K}_{3} (t,{\mathcal{T}}(t),{\mathcal{H}}(t),{ \mathcal{E}}(t) )= \frac{k_{3} \mathcal{T}(t)\mathcal{E}(t)}{\mathcal{T}(t)+s_{3}}- \beta _{31} \mathcal{T}(t)\mathcal{E}(t)-c_{3} \mathcal{E}(t). \end{cases} $$

#### Theorem 2

*The initial value problem and the kernels* $\mathcal{K}_{1} ({\mathcal{T}}(t),{\mathcal{H}}(t),{ \mathcal{E}}(t) )$, $\mathcal{K}_{2} ({\mathcal{T}}(t),{\mathcal{H}}(t), { \mathcal{E}}(t) )$, *and* $\mathcal{K}_{3} ({\mathcal{T}}(t),{\mathcal{H}}(t),{ \mathcal{E}}(t) )$ *satisfy the Lipschitz condition*.

#### Proof

See [24]. □

#### Theorem 3

*The fractional nonlinear system* (29) *admits at least one solution*.

#### Proof

See [24]. □

#### Theorem 4

*The fractional nonlinear system* (29) *always admits a unique solution*.

#### Proof

See [24]. □

### Numerical method

Now let us focus on determining an approximate solution to the following CFC fractional Cauchy problem:

35 $$ {}^{\mathsf{CFC}}_{0}{\mathcal{D}}_{t}^{{\boldsymbol{\alpha }}}\phi (t)=\Xi \bigl(t, \phi (t)\bigr). $$

Using the corresponding fractional integral operator yields

36 $$ \phi (t) - \phi (0) = \frac{1 - {\boldsymbol{\alpha }}}{\mathsf{M}({\boldsymbol{\alpha }})} \Xi \bigl(t,\phi (t) \bigr) + \frac{{\boldsymbol{\alpha }}}{\mathsf{M}({\boldsymbol{\alpha }})} \int _{0}^{t}\Xi \bigl({ \eta },\phi ({\eta }) \bigr)\, d{\eta }. $$

Taking $t = t_{n+1}$ in (36), we have

37 $$ \phi (t_{n+1}) - \phi (0) = \frac{(2 - {\boldsymbol{\alpha }})(1-{\boldsymbol{\alpha }})}{2}\Xi \bigl(t_{n},\phi (t_{n})\bigr) + \frac{{\boldsymbol{\alpha }}(2-{\boldsymbol{\alpha }})}{2} \int _{0}^{t_{n+1}} \Xi \bigl({ \eta },\phi ({\eta }) \bigr)\, d{\eta } $$

and

38 $$ \phi (t_{n}) - \phi (0) = \frac{(2-{\boldsymbol{\alpha }})(1-{\boldsymbol{\alpha }})}{2}\Xi \bigl(t_{n-1},\phi (t_{n-1})\bigr) + \frac{{\boldsymbol{\alpha }}(2-{\boldsymbol{\alpha }})}{2} \int _{0}^{t_{n}} \Xi \bigl({ \eta },\phi ({\eta }) \bigr)\, d{\eta }. $$

Inserting Eq. (38) into Eq. (37), we get

39 $$ \begin{aligned}[b] \phi (t_{n+1}) &= \phi (t_{n}) + \frac{(2-{\boldsymbol{\alpha }})(1-{\boldsymbol{\alpha }})}{2} \bigl[ \Xi \bigl(t_{n},\phi (t_{n})\bigr) - \Xi \bigl(t_{n-1},\phi (t_{n-1})\bigr) \bigr] \\ &\quad {}+ \frac{{\boldsymbol{\alpha }}(2-{\boldsymbol{\alpha }})}{2} \int _{t_{n}}^{t_{n+1}} \Xi \bigl({ \eta },\phi ({\eta }) \bigr)\, d{\eta }, \end{aligned} $$

where

40 $$ \begin{aligned} \int _{t_{n}}^{t_{n+1}}\Xi \bigl({\eta },\phi ({\eta }) \bigr)\, d{\eta } & = \frac{3\hbar }{2}\Xi (t_{n},f_{n}) - \frac{\hbar }{2}\Xi (t_{n-1},f_{n-1}). \end{aligned} $$

So we have

41 $$ \begin{aligned}[b] f_{n+1} &= f_{n} + \biggl[ \frac{(2 - {\boldsymbol{\alpha }})(1 - {\boldsymbol{\alpha }})}{2} + \frac{3\hbar }{4}{ \boldsymbol{\alpha }}(2 - { \boldsymbol{\alpha }}) \biggr]\Xi (t_{n},f_{n}) \\ &\quad {}- \biggl[ \frac{(2 - {\boldsymbol{\alpha }})(1 - {\boldsymbol{\alpha }})}{2} + \frac{\hbar }{4}{ \boldsymbol{\alpha }}(2 - {\boldsymbol{ \alpha }}) \biggr]\Xi (t_{n-1},f_{n-1}). \end{aligned} $$

As a result, the following recursive relations are determined to approximate the CFC problem (29) as in [22]:

42 $$ \begin{gathered} \mathcal{T}_{{n+1}}(t) = \mathcal{T}(0) + \biggl[ \frac{(2 - {\boldsymbol{\alpha }})(1 - {\boldsymbol{\alpha }})}{2} + \frac{3\hbar }{4}{ \boldsymbol{ \alpha }}(2 - {\boldsymbol{\alpha }}) \biggr]\Xi _{1}\bigl( \mathcal{T}_{{n}}(t), \mathcal{H}_{{n}}(t),\mathcal{E}_{{n}}(t),t_{n} \bigr) \\ \hphantom{\mathcal{T}_{{n+1}}(t) =} {} - \biggl[ \frac{(2 - {\boldsymbol{\alpha }})(1 - {\boldsymbol{\alpha }})}{2} + \frac{\hbar }{4}{\boldsymbol{\alpha }}(2-{\boldsymbol{\alpha }}) \biggr]\Xi _{1}\bigl( \mathcal{T}_{{n}}(t),\mathcal{H}_{{n}}(t),\mathcal{E}_{{n}}(t),t_{n} \bigr), \\ \mathcal{H}_{{n+1}}(t) = \mathcal{H}(0) + \biggl[ \frac{(2 - {\boldsymbol{\alpha }})(1 - {\boldsymbol{\alpha }})}{2} + \frac{3\hbar }{4}{ \boldsymbol{\alpha }}(2 - {\boldsymbol{ \alpha }}) \biggr]\Xi _{2}\bigl(\mathcal{T}_{{n}}(t), \mathcal{H}_{{n}}(t),\mathcal{E}_{{n}}(t),t_{n}\bigr) \\ \hphantom{\mathcal{H}_{{n+1}}(t) =} {} - \biggl[ \frac{(2 - {\boldsymbol{\alpha }})(1 - {\boldsymbol{\alpha }})}{2} + \frac{\hbar }{4}{\boldsymbol{\alpha }}(2-{\boldsymbol{\alpha }}) \biggr]\Xi _{2}\bigl( \mathcal{T}_{{n}}(t),\mathcal{H}_{{n}}(t),\mathcal{E}_{{n}}(t),t_{n} \bigr), \\ \mathcal{E}_{{n+1}}(t) = \mathcal{E}(0) + \biggl[ \frac{(2 - {\boldsymbol{\alpha }})(1 - {\boldsymbol{\alpha }})}{2} + \frac{3\hbar }{4}{ \boldsymbol{\alpha }}(2-{\boldsymbol{ \alpha }}) \biggr]\Xi _{3}\bigl(\mathcal{T}_{{n}}(t), \mathcal{H}_{{n}}(t),\mathcal{E}_{{n}}(t),t_{n}\bigr) \\ \hphantom{\mathcal{E}_{{n+1}}(t) =} {} - \biggl[ \frac{(2 - {\boldsymbol{\alpha }})(1 - {\boldsymbol{\alpha }})}{2} + \frac{\hbar }{4}{\boldsymbol{\alpha }}(2-{\boldsymbol{\alpha }}) \biggr]\Xi _{3}\bigl( \mathcal{T}_{{n}}(t),\mathcal{H}_{{n}}(t),\mathcal{E}_{{n}}(t),t_{n} \bigr), \end{gathered} $$

where $\Xi _{1}= \mathcal{T} (t) (1-\mathcal{T}(t) )-\beta _{12} \mathcal{T}(t)\mathcal{H}(t)-\beta _{13} \mathcal{T}(t)\mathcal{E}(t)$, $\Xi _{2}=k_{2} \mathcal{H} (t) (1-\mathcal{H}(t) )- \beta _{21} \mathcal{T}(t)\mathcal{H}(t)$, and $\Xi _{3}= \frac{k_{3} \mathcal{T}(t)\mathcal{E}(t)}{\mathcal{T}(t)+s_{3}}-a_{31} \mathcal{T}(t)\mathcal{E}(t)-c_{3} \mathcal{E}(t)$.

### Numerical simulations

**Figure 4:**
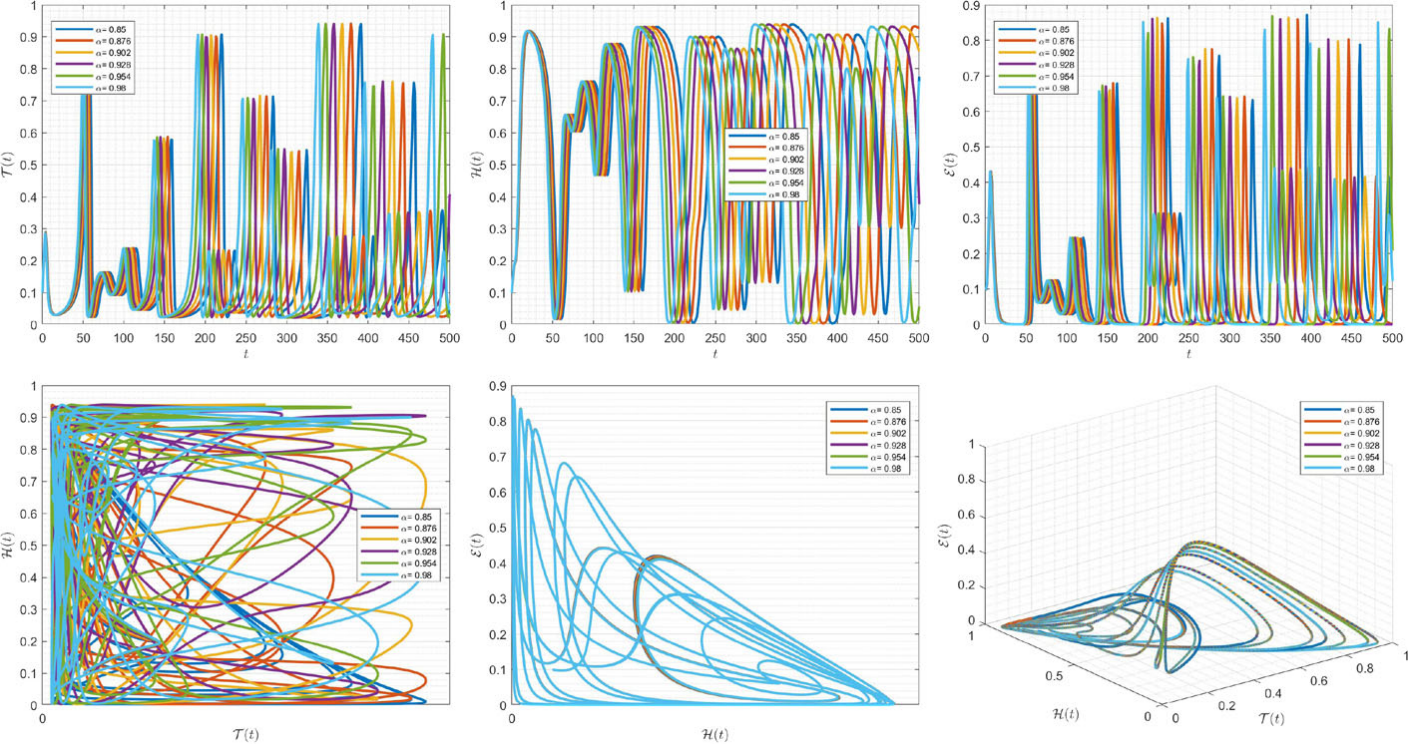
Simulations for solving (29) using (42) along with $(\mathcal{T}(0),\mathcal{H}(0),\mathcal{E}(0) )= (0.1,0.1,0.1 )$

**Figure 5:**
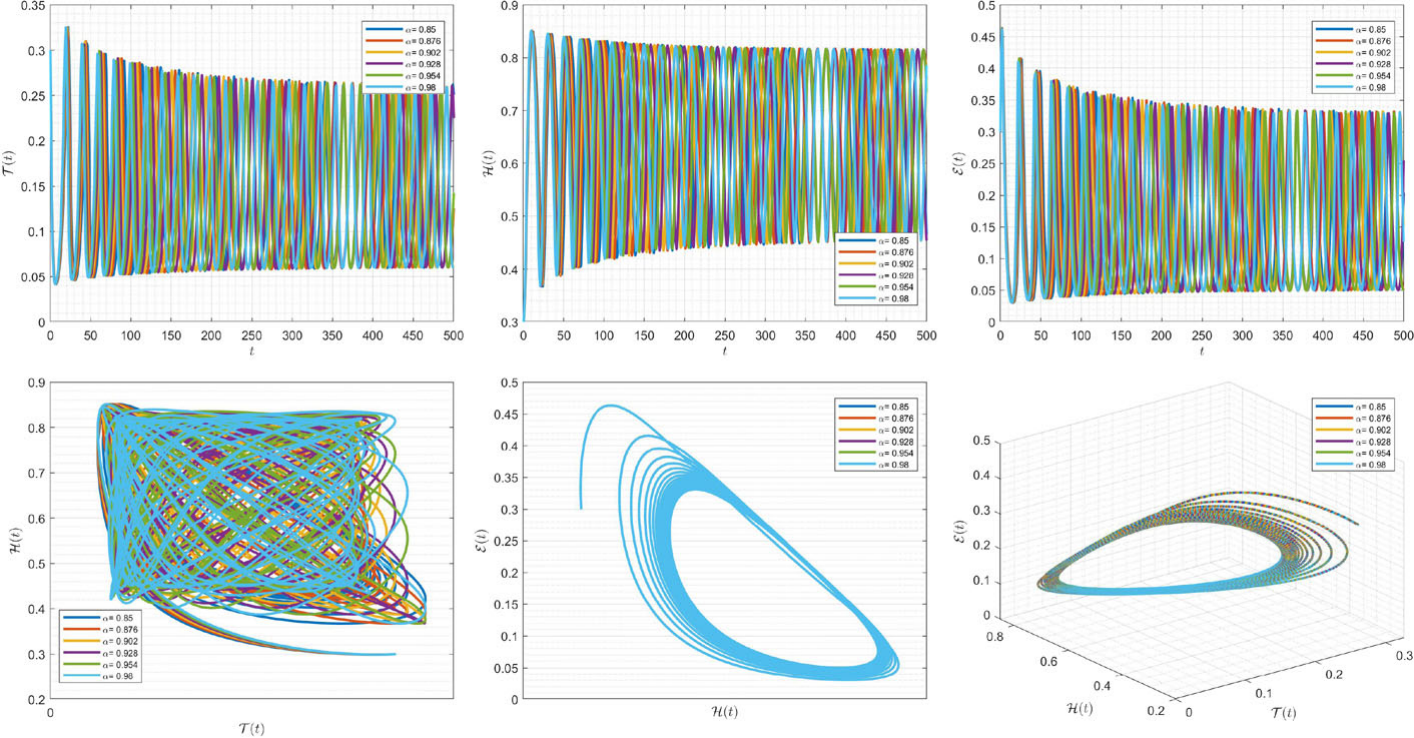
Simulations for solving (29) using (42) along with $(\mathcal{T}(0),\mathcal{H}(0),\mathcal{E}(0) )= (0.3,0.3,0.3 )$ and $\beta _{12}= 0.745$

**Figure 6:**
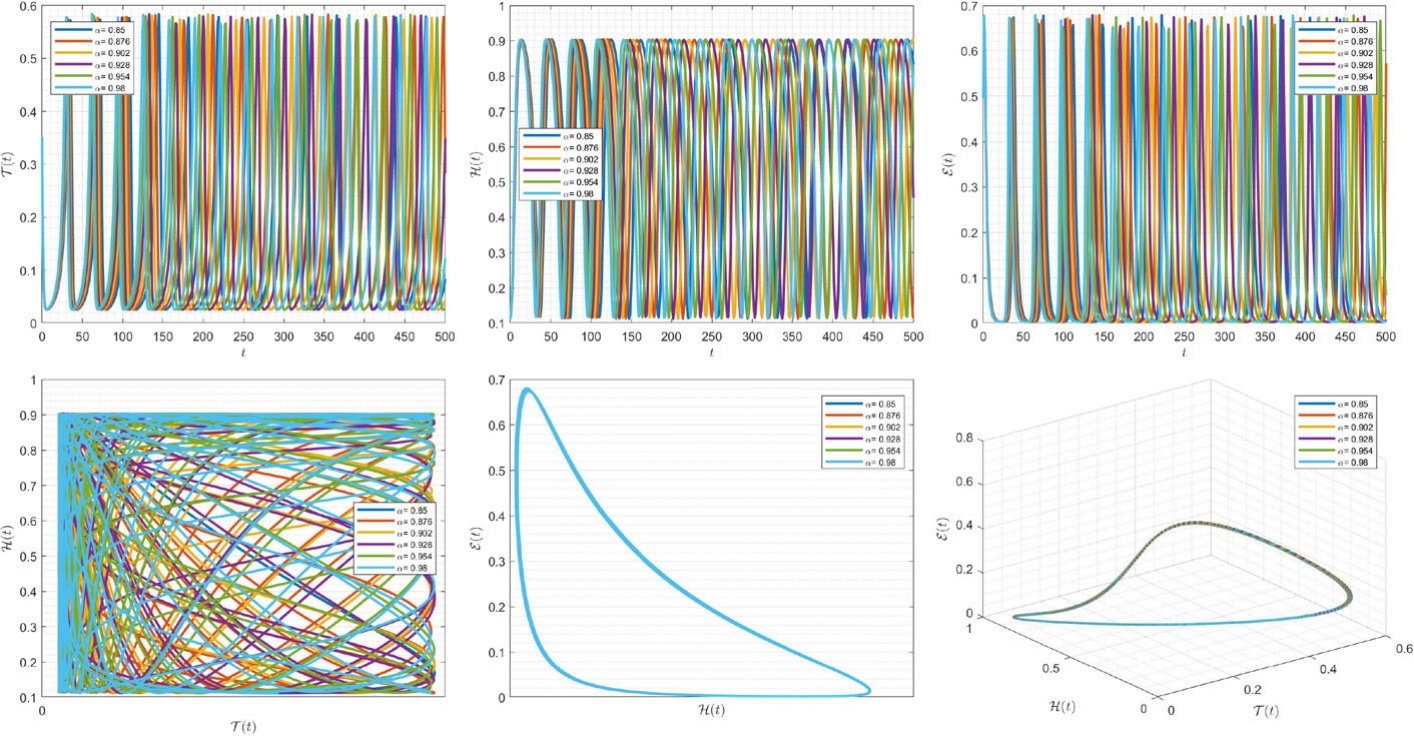
Simulations for solving (29) using (42) along with $(\mathcal{T}(0),\mathcal{H}(0),\mathcal{E}(0) )= ( 0.3517, 0.1115, 0.4951 )$ and $\beta _{12}= 0.92$

Figures 4–6 are plotted to demonstrate the variation of state variables in model (29) when the scheme (42) is employed for different values of $\alpha \in (0,1]$. In these simulations, we considered the following values in the model: $\beta _{12}=1$, $\beta _{13}=2.5$, $k_{2}=0.6$, $\beta _{21}=1.5$, $k_{3}=4.5$, $s_{3}=1$, $a_{31}=0.2$, and $d_{3}=0.5$. In our performed numerical simulations, we considered $t_{\mathrm{final}}= 500$ and $\hbar = 0.001$. In Fig. 4, we use $(\mathcal{T}(t), \mathcal{H}(t), \mathcal{E}(t) )|_{t=0}= (0.1, 0.1, 0.1 )$. In this case the model exhibits chaotic attractor behavior. Also, starting from $(\mathcal{T}(t), \mathcal{H}(t), \mathcal{E}(t) )|_{t=0}= (0.3, 0.3, 0.3 )$ and $\beta _{12} = 0.745 $, the model shows the limit cycle behavior in Fig. 5. Further, by taking $\mathcal{T}(0)=0.3517$, $\mathcal{H}(0)=0.1115$, $\mathcal{E}(0)=0.4951$, and $\beta _{12} = 0.920$, we get periodic orbit trajectories in the solutions as depicted in Fig. 6.

## The model via Atangana–Baleanu–Caputo fractional derivative

Now let us consider the model via the Atangana–Baleanu–Caputo fractional derivative as

43 $$\begin{aligned}& {}_{0}^{\mathsf{ABC}} \mathcal{D}_{t}^{{\boldsymbol{\alpha }}} {\mathcal{T}}(t)= \mathcal{T} (t) \bigl(1- \mathcal{T}(t) \bigr)-\beta _{12} { \mathcal{T}}(t)\mathcal{H}(t)-\beta _{13} {\mathcal{T}}(t)\mathcal{E}(t), \\& {}_{0}^{\mathsf{ABC}} \mathcal{D}_{t}^{{\boldsymbol{\alpha }}} { \mathcal{H}}(t)=k_{2} \mathcal{H} (t) \bigl(1-\mathcal{H}(t) \bigr)- \beta _{21} \mathcal{T}(t)\mathcal{H}(t), \\& {}_{0}^{\mathsf{ABC}} \mathcal{D}_{t}^{{\boldsymbol{\alpha }}} { \mathcal{E}}(t)= \frac{k_{3} \mathcal{T}(t)\mathcal{E}(t)}{\mathcal{T}(t)+s_{3}}- \beta _{31} \mathcal{T}(t) \mathcal{E}(t)-c_{3} \mathcal{E}(t), \end{aligned}$$

where the Atangana–Baleanu fractional integral of order ***α*** of a function $\phi (t)$ is defined as [10]

44 $$ {}_{0}^{\mathsf{ABC}} \mathcal{D}_{t}^{{\boldsymbol{\alpha }}} {\phi }(t)= \frac{\mathsf{B}({\boldsymbol{\alpha }})}{ n-{{{\boldsymbol{\alpha }}}}} \int _{0}^{t} \phi '({\eta } )E_{\boldsymbol{\alpha }} \biggl[- \frac{{\boldsymbol{\alpha }}}{n-{\boldsymbol{\alpha }}}(t-{\eta })^{\boldsymbol{\alpha }} \biggr] d{ \eta }, $$

where $\mathsf{B}({\boldsymbol{\alpha }})=1-\alpha +\frac{\alpha }{\Gamma (\alpha )}$ is a normalization function.

The Atangana–Baleanu fractional integral of order ***α*** of a function $\phi (t)$ is also defined as [10]

45 $$ {}_{0}^{\mathsf{ABC}} \mathcal{I}_{t}^{{\boldsymbol{\alpha }}} {\phi }(t)= \frac{1-{\boldsymbol{\alpha }}}{\mathsf{B}({\boldsymbol{\alpha }})}\phi (t)+ \frac{{\boldsymbol{\alpha }}}{\mathsf{B}({\boldsymbol{\alpha }})\Gamma ({\boldsymbol{\alpha }})} \int _{0}^{t}\phi ({\eta }) (t-{\eta })^{{\boldsymbol{\alpha }}-1}\,d{\eta }. $$

Taking the definition of the Atangana–Baleanu fractional integral (45) on both sides of equations in system (43), we get the Volterra integral system

46 $$ \begin{gathered} \mathcal{T}(t)-\mathcal{T}(0)\\ \quad = \frac{ 1-{\boldsymbol{\alpha }} }{ \mathsf{B}({\boldsymbol{\alpha }} )}\bigl\{ \mathcal{T} (t) \bigl(1-\mathcal{T}(t) \bigr)-\beta _{12} \mathcal{T}(t)\mathcal{H}(t)- \beta _{13} \mathcal{T}(t)\mathcal{E}(t) \bigr\} \\ \qquad {}+ \frac{ {\boldsymbol{\alpha }} }{\mathsf{B}({\boldsymbol{\alpha }})\Gamma ({\boldsymbol{\alpha }})} \int _{0}^{t}\bigl\{ \mathcal{T} ({\eta }) \bigl(1- \mathcal{T}({\eta }) \bigr)-\beta _{12} \mathcal{T}({\eta }) \mathcal{H}({\eta })-\beta _{13} \mathcal{T}(t)\mathcal{E}(t) \bigr\} (t-{\eta })^{{\boldsymbol{\alpha }}-1}\,d{\eta }, \\ \mathcal{H}(t)-\mathcal{H}(0)\\ \quad = \frac{ 1-{\boldsymbol{\alpha }} }{ \mathsf{B}({\boldsymbol{\alpha }} )}\bigl\{ k_{2} \mathcal{H} (t) \bigl(1-\mathcal{H}(t) \bigr)-\beta _{21} \mathcal{T}(t) \mathcal{H}(t) \bigr\} \\ \qquad {}+ \frac{ {\boldsymbol{\alpha }} }{\mathsf{B}({\boldsymbol{\alpha }})\Gamma ({\boldsymbol{\alpha }})} \int _{0}^{t}\bigl\{ k_{2} \mathcal{H} ({ \eta }) \bigl(1-\mathcal{H}({\eta }) \bigr)-\beta _{21} \mathcal{T}({ \eta })\mathcal{H}({\eta }) \bigr\} (t-{ \eta })^{{\boldsymbol{\alpha }}-1}\,d{\eta }, \\ \mathcal{E}(t)-\mathcal{E}(0)\\ \quad = \frac{ 1-{\boldsymbol{\alpha }} }{ \mathsf{B}({\boldsymbol{\alpha }} )}\biggl\{ \frac{k_{3} \mathcal{T}(t)\mathcal{E}(t)}{\mathcal{T}(t)+s_{3}}- \beta _{31} \mathcal{T}(t)\mathcal{E}(t)-c_{3} \mathcal{E}(t) \biggr\} \\ \qquad {}+ \frac{ {\boldsymbol{\alpha }} }{\mathsf{B}({\boldsymbol{\alpha }})\Gamma ({\boldsymbol{\alpha }})} \int _{0}^{t}\biggl\{ \frac{k_{3} \mathcal{T}({\eta })\mathcal{E}({\eta })}{\mathcal{T}({\eta })+s_{3}}- \beta _{31} \mathcal{T}({\eta })\mathcal{E}({\eta })-c_{3} \mathcal{E}({ \eta }) \biggr\} \,d {\eta } . \end{gathered} $$

and the following iterative formulas:

47 $$\begin{aligned}& \begin{gathered} \mathcal{T}_{n+1}(t)\\ \quad = \mathcal{T}(0)+ \frac{ 1-{\boldsymbol{\alpha }} }{ \mathsf{B}({\boldsymbol{\alpha }} )}\bigl\{ \mathcal{T}_{n} (t) \bigl(1- \mathcal{T}_{n} (t) \bigr)-\beta _{12} \mathcal{T}_{n} (t) \mathcal{H}_{n} (t)-\beta _{13} \mathcal{T}_{n} (t)\mathcal{E}_{n} (t) \bigr\} \} \\ \qquad + \frac{ {\boldsymbol{\alpha }} }{\mathsf{B}({\boldsymbol{\alpha }})\Gamma ({\boldsymbol{\alpha }})} \int _{0}^{t}\bigl\{ \mathcal{T}_{n} ({ \eta }) \bigl(1-\mathcal{T}_{n} ({ \eta }) \bigr)-\beta _{12} \mathcal{T}_{n} ({\eta })\mathcal{H}_{n} ({ \eta })-\beta _{13} \mathcal{T}_{n} ({\eta })\mathcal{E}_{n} ({ \eta }) \bigr\} (t-{\eta })^{{\boldsymbol{\alpha }}-1}\,d{\eta }, \end{gathered} \\& \begin{gathered} \mathcal{H}_{n+1}(t)\\ \quad =\mathcal{H}(0)+ \frac{ 1-{\boldsymbol{\alpha }} }{ \mathsf{B}({\boldsymbol{\alpha }} )}\bigl\{ k_{2} \mathcal{H}_{n} (t) \bigl(1-\mathcal{H}_{n}(t) \bigr)-\beta _{21} \mathcal{T}_{n}(t)\mathcal{H}_{n}(t) \bigr\} \\ \qquad {}+ \frac{ {\boldsymbol{\alpha }} }{\mathsf{B}({\boldsymbol{\alpha }})\Gamma ({\boldsymbol{\alpha }})} \int _{0}^{t}\bigl\{ k_{2} \mathcal{H}_{n} ({\eta }) \bigl(1-\mathcal{H}_{n}({ \eta }) \bigr)-\beta _{21} \mathcal{T}_{n}({\eta }) \mathcal{H}_{n}({ \eta }) \bigr\} (t-{\eta })^{{\boldsymbol{\alpha }}-1}\,d{\eta }, \end{gathered} \\& \begin{gathered} \mathcal{E}_{n+1}(t)\\ \quad =\mathcal{E}(0)+ \frac{ 1-{\boldsymbol{\alpha }} }{ \mathsf{B}({\boldsymbol{\alpha }} )}\biggl\{ \frac{k_{3} \mathcal{T}_{n}(t)\mathcal{E}_{n}(t)}{\mathcal{T}_{n}(t)+s_{3}}- \beta _{31} \mathcal{T}_{n}(t) \mathcal{E}_{n}(t)-c_{3} \mathcal{E}_{n}(t) \biggr\} \\ \qquad {}+\frac{ {\boldsymbol{\alpha }} }{\mathsf{B}({\boldsymbol{\alpha }})\Gamma ({\boldsymbol{\alpha }})} \int _{0}^{t}\biggl\{ \frac{k_{3} \mathcal{T}_{n}({\eta })\mathcal{E}_{n}({\eta })}{\mathcal{T}_{n}({\eta })+s_{3}}- \beta _{31} \mathcal{T}_{n}({\eta })\mathcal{E}_{n}({ \eta })-c_{3} \mathcal{E}_{n}({\eta }) \biggr\} (t-{\eta })^{{\boldsymbol{\alpha }}-1}\,d{\eta }, \end{gathered} \end{aligned}$$

where

48 $$ \mathcal{T}_{0}(t)= \mathcal{T}(0),\qquad \mathcal{H}_{0}(t)= \mathcal{H}(0),\qquad \mathcal{E}_{0}(t)= \mathcal{E}(0). $$

As $n \rightarrow \infty $, Eq. (47) suggests the exact solution for the model.

### Theorem 5

*The initial value problem given by Eq*. (43) *possesses at least one solution in the interval* $[0, T]$.

### Proof

First, we define

49 $$ \textstyle\begin{cases} \mathcal{E}_{1}(t,\mathcal{T}(t))= \mathcal{T} (t) (1- \mathcal{T}(t) )-\beta _{12} \mathcal{T}(t)\mathcal{H}(t)- \beta _{13} \mathcal{T}(t)\mathcal{E}(t), \\ \mathcal{E}_{2}(t,\mathcal{H}(t)) =k_{2} \mathcal{H} (t) (1- \mathcal{H}(t) )-\beta _{21} \mathcal{T}(t)\mathcal{H}(t), \\ \mathcal{E}_{3}(t,\mathcal{E}(t))= \frac{k_{3} \mathcal{T}(t)\mathcal{E}(t)}{\mathcal{T}(t)+s_{3}}- \beta _{31} \mathcal{T}(t)\mathcal{E}(t)-c_{3} \mathcal{E}(t), \end{cases} $$

where $\mathcal{E}_{1}(t,\mathcal{T}(t))$, $\mathcal{E}_{2}(t,\mathcal{H}(t))$, and $\mathcal{E}_{3}(t,\mathcal{E}(t))$ are contractions respect to $\mathcal{T}(t)$, $\mathcal{H}(t)$, and $\mathcal{E}(t)$, respectively.

Moreover, we set

50 $$ \begin{gathered} \mathcal{N}_{1}= \sup \bigl\Vert {}_{\Psi _{a,b_{1}}} \mathcal{E}_{1}\bigl(t, \mathcal{T}(t)\bigr) \bigr\Vert ,\qquad \mathcal{N}_{2}= \sup \bigl\Vert {}_{\Psi _{a,b_{2}}} \mathcal{E}_{2}\bigl(t,\mathcal{H}(t)\bigr) \bigr\Vert ,\\ \mathcal{N}_{3}= \sup \bigl\Vert _{ \Psi _{a,b_{3}}} \mathcal{E}_{3}\bigl(t,\mathcal{E}(t)\bigr) \bigr\Vert , \end{gathered} $$

where

51 $$ \textstyle\begin{cases} \Psi _{a,b_{1}}= [t-a,t+a ]\times [\mathcal{T}-b_{1}, \mathcal{T}+b_{1} ]=A_{1}\times B_{1}, \\ \Psi _{a,b_{2}}= [t-a,t+a ]\times [\mathcal{T}-b_{2}, \mathcal{H}+b_{2} ]=A_{1}\times B_{2}, \\ \Psi _{a,b_{3}}= [t-a,t+a ]\times [\mathcal{T}-b_{3}, \mathcal{E}+b_{3} ]=A_{1}\times B_{3}. \end{cases} $$

Considering the Picard operator, we have

52 $$ \Theta : (\Psi _{a,b_{1}},\Psi _{a,b_{2}},\Psi _{a,b_{3}} ) \rightarrow (\Psi _{a,b_{1}},\Psi _{a,b_{2}},\Psi _{a,b_{3}} ) $$

defined as follows

53 $$ \Theta \Omega (t)=\Omega _{0}(t)+ \frac{ 1-{\boldsymbol{\alpha }} }{ \mathsf{B}({\boldsymbol{\alpha }} )}\Delta \bigl(t, \Omega (t)\bigr) + \frac{ {\boldsymbol{\alpha }} }{\mathsf{B}({\boldsymbol{\alpha }})\Gamma ({\boldsymbol{\alpha }})} \int _{0}^{t}\Delta \bigl({\eta },\Omega ({\eta }) \bigr) (t-{\eta })^{{\boldsymbol{\alpha }}-1}\,d{ \eta }, $$

where $\Omega (t) = ({\mathcal{T}(t), \mathcal{H}(t), \mathcal{E}(t) } )$, $\Omega _{0}(t) = ({\mathcal{T}(0), \mathcal{H}(0), \mathcal{E}(0)} )$, and $\Delta (t, \Omega (t)) = \mathcal{E}_{1}(t,\mathcal{T}(t)), \mathcal{E}_{2}(t,\mathcal{H}(t)),\mathcal{E}_{3}(t,\mathcal{E}(t)) $.

Now we assume that all the solutions are bounded within a period of time:

54 $$ \begin{gathered} \bigl\Vert \Omega (t) \bigr\Vert _{\infty }\le \max \{B_{1},B_{2},B_{3},B_{4} \}, \\ \bigl\Vert \Omega (t)-\Omega _{0}(t) \bigr\Vert _{\infty } \\ \quad \le \biggl\| \frac{ 1-{\boldsymbol{\alpha }} }{ \mathsf{B}({\boldsymbol{\alpha }} )}\Delta \bigl(t, \Omega (t)\bigr) + \frac{ {\boldsymbol{\alpha }} }{\mathsf{B}({\boldsymbol{\alpha }})\Gamma ({\boldsymbol{\alpha }})} \int _{0}^{t}\Delta \bigl({\eta },\Omega ({\eta }) \bigr) (t-{\eta })^{{\boldsymbol{\alpha }}-1}\,d{ \eta }\biggr\| \\ \quad \le \frac{ 1-{\boldsymbol{\alpha }} }{ \mathsf{B}({\boldsymbol{\alpha }} )} \bigl\Vert \Delta \bigl(t, \Omega (t)\bigr) \bigr\Vert + \frac{ {\boldsymbol{\alpha }} }{\mathsf{B}({\boldsymbol{\alpha }})\Gamma ({\boldsymbol{\alpha }})} \int _{0}^{t}\bigl\| \Delta \bigl({\eta },\Omega ({\eta }) \bigr)\bigr\| (t-{\eta })^{{\boldsymbol{\alpha }}-1}\,d{\eta } \\ \quad \le \frac{ 1-{\boldsymbol{\alpha }} }{ \mathsf{B}({\boldsymbol{\alpha }} )\Gamma ({\boldsymbol{\alpha }})}Z=\max \{B_{1}, B_{2},B_{3},B_{4} \}+ \frac{ {\boldsymbol{\alpha }} }{\mathsf{B}({\boldsymbol{\alpha }})\Gamma ({\boldsymbol{\alpha }})}Z \omega ^{\boldsymbol{\alpha }}\\ \quad \le \omega Z< B=\max \{B_{1}, B_{2},B_{3},B_{4} \}, \end{gathered} $$

provided that

$$ \omega < \frac{B}{Z}. $$

The fixed point theorem of a Banach space together with the metric suggests that

55 $$ \begin{gathered} \Vert \Theta \Omega _{1}-\Theta \Omega _{2} \Vert _{\infty }= \sup_{t\in A} \vert \Omega _{1}-\Omega _{2} \vert , \\ \Vert \Theta \Omega _{1}-\Theta \Omega _{2} \Vert \\ \quad = \bigl\Vert \Delta \bigl(t,\Omega _{1}(t)\bigr)- \Delta \bigl(t, \Omega _{2}(t)\bigr)\bigr\| \frac{ 1-{\boldsymbol{\alpha }} }{ \mathsf{B}({\boldsymbol{\alpha }} )} \\ \qquad {}+ \frac{ {\boldsymbol{\alpha }} }{\mathsf{B}({\boldsymbol{\alpha }})\Gamma ({\boldsymbol{\alpha }})} \int _{0}^{t}\bigl\{ \Delta \bigl(t,\Omega _{1}(t)\bigr)-\Delta \bigl(t,\Omega _{2}(t)\bigr)\bigr\} (t-{ \eta })^{{\boldsymbol{\alpha }}-1}\,d{\eta } \Vert \\ \quad \le \frac{ 1-{\boldsymbol{\alpha }} }{ \mathsf{B}({\boldsymbol{\alpha }} )} \bigl\Vert \Delta \bigl(t, \Omega _{1}(t) \bigr)-\Delta \bigl(t,\Omega _{2}(t)\bigr) \bigr\Vert \\ \qquad {}+ \frac{ {\boldsymbol{\alpha }} }{\mathsf{B}({\boldsymbol{\alpha }})\Gamma ({\boldsymbol{\alpha }})} \int _{0}^{t} \bigl\Vert \Delta \bigl(t,\Omega _{1}(t)\bigr)-\Delta \bigl(t,\Omega _{2}(t)\bigr) \bigr\Vert (t-{ \eta })^{{\boldsymbol{\alpha }}-1}\,d{\eta } \\ \quad \le \frac{ 1-{\boldsymbol{\alpha }} }{ \mathsf{B}({\boldsymbol{\alpha }} )}{\eta } \bigl\Vert \Omega _{1}(t)-\Omega _{2}(t) \bigr\Vert \\ \qquad {}+ \frac{ {\boldsymbol{\alpha }} {\eta }}{\mathsf{B}({\boldsymbol{\alpha }})\Gamma ({\boldsymbol{\alpha }})} \int _{0}^{t} \bigl\Vert \Omega _{1}(t)-\Omega _{2}(t) \bigr\Vert (t-{\eta })^{{\boldsymbol{\alpha }}-1}\,d{\eta } \\ \quad \le \biggl\{ \frac{ 1-{\boldsymbol{\alpha }} }{ \mathsf{B}({\boldsymbol{\alpha }} )}{\eta }+ \frac{ {\boldsymbol{\alpha }} {\eta }}{\mathsf{B}({\boldsymbol{\alpha }})\Gamma ({\boldsymbol{\alpha }})} \biggl( \frac{a^{\boldsymbol{\alpha }}}{{\boldsymbol{\alpha }}}\biggr)\biggr\} \bigl\Vert \Omega _{1}(t)-\Omega _{2}(t) \bigr\Vert \\ \quad \le {\eta } \omega \bigl\Vert \Omega _{1}(t)-\Omega _{2}(t) \bigr\Vert . \end{gathered} $$

Since Ω is a contraction along with ${\eta } < 1$, we must have $\omega {\eta } < 1$. Therefore we conclude that Θ is a contraction operator. Finally, it is proved that (43) always possesses a unique solution. □

### Numerical method

Consider the following fractional initial value problem:

56 $$ \begin{aligned} &{}_{0}^{\mathsf{ABC}} \mathcal{D}_{t}^{{\boldsymbol{\alpha }}} {\phi }(t) = \Xi \bigl(t, \phi (t)\bigr), \\ &\phi (0)=\phi _{0}. \end{aligned} $$

Employing the product integration rule, Ghanbari and Kumar [20] have developed an efficient scheme to obtain the approximate solution of (56), given by

57 $$ \begin{aligned} \phi _{n}&= \phi _{0} + \frac{\alpha \hslash ^{\alpha }}{\mathsf{B}(\alpha )} \Biggl(p_{n} \Xi (t_{0}, \phi _{0} )+\sum_{\zeta =1}^{n}q_{-\zeta +n} \Xi (t_{\zeta }, \phi _{ \zeta } ) \Biggr),\quad n\ge 1, \end{aligned} $$

where

58 $$ \begin{aligned} p_{n}&= \frac{(n-1)^{\alpha +1}-n^{\alpha }(n-\alpha -1)}{\Gamma (\alpha +2)}, \\ q_{j}&=\textstyle\begin{cases} \frac{1}{{\Gamma (\alpha + 2)}}+ \frac{1-\alpha }{\alpha \hslash ^{\alpha }},& j = 0, \\ \frac{{{{(j- 1)}^{\alpha +1} } - 2{j^{\alpha +1}} + {{(j + 1)}^{\alpha +1}}}}{{\Gamma (\alpha + 2)}}, & j = 1,2, \ldots ,n-1. \end{cases}\displaystyle \end{aligned} $$

Using this numerical approximation, we get the following iterative scheme:

59 $$ \begin{gathered} \mathcal{T}_{ n} = \mathcal{T}_{ 0} + \frac{{\alpha }\hslash ^{\alpha }}{\mathsf{B}({\alpha })} \Biggl({p}_{n} \bigl[ \mathcal{T}_{0} (1-\mathcal{T}_{0} )-\beta _{12} \mathcal{T}_{0}\mathcal{H}_{0}-\beta _{13} \mathcal{T}_{0}\mathcal{E}_{0} \bigr]\\ \hphantom{\mathcal{T}_{ n} =}{} +\sum _{\zeta =1}^{n}{q}_{n-i} \bigl[ \mathcal{T}_{\zeta } (1-\mathcal{T}_{\zeta } )-\beta _{12} \mathcal{T}_{\zeta } \mathcal{H}_{\zeta }-\beta _{13} \mathcal{T}_{\zeta }\mathcal{E}_{\zeta } \bigr] \Biggr), \\ \mathcal{H}_{n} = \mathcal{H}_{0} + \frac{{\alpha }\hslash ^{\alpha }}{\mathsf{B}({\alpha })} \Biggl({p}_{n} \bigl[k_{2} \mathcal{H}_{0} (1- \mathcal{H}_{0} )-\beta _{21} \mathcal{T}_{0} \mathcal{H}_{0} \bigr]\\ \hphantom{\mathcal{H}_{n} =}{} +\sum_{\zeta =1}^{n}{q}_{n-i} \bigl[k_{2} \mathcal{H}_{\zeta } (1-\mathcal{H}_{\zeta } )- \beta _{21} \mathcal{T}_{\zeta }\mathcal{H}_{\zeta } \bigr] \Biggr), \\ \mathcal{E}_{ n} = \mathcal{E}_{ 0} + \frac{{\alpha }\hslash ^{\alpha }}{\mathsf{B}({\alpha })} \Biggl({p}_{n} \biggl[ \frac{k_{3} \mathcal{T}_{0}\mathcal{E}_{0}}{\mathcal{T}_{0}+s_{3}}- \beta _{31} \mathcal{T}_{0}\mathcal{E}_{0}-c_{3} \mathcal{E}_{0} \biggr]\\ \hphantom{\mathcal{E}_{ n} =}{}+\sum_{\zeta =1}^{n}{q}_{n-i} \biggl[ \frac{k_{3} \mathcal{T}_{\zeta }\mathcal{E}_{\zeta }}{\mathcal{T}_{\zeta }+s_{3}}- \beta _{31} \mathcal{T}_{\zeta } \mathcal{E}_{\zeta }-c_{3} \mathcal{E}_{ \zeta } \biggr] \Biggr). \end{gathered} $$

### Numerical simulations

**Figure 7:**
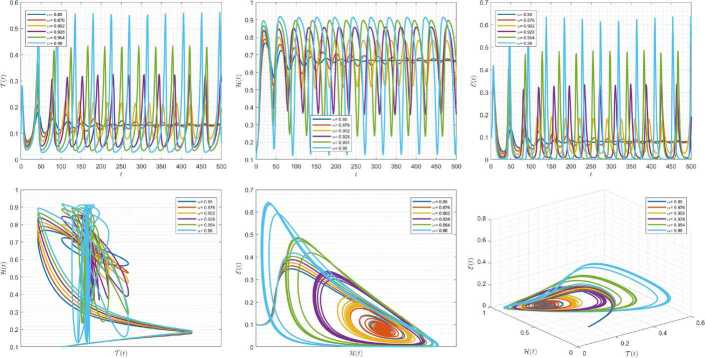
Simulations for solving (43) using (59) along with $(\mathcal{T}(0),\mathcal{H}(0),\mathcal{E}(0) )= (0.1,0.1,0.1 )$

**Figure 8:**
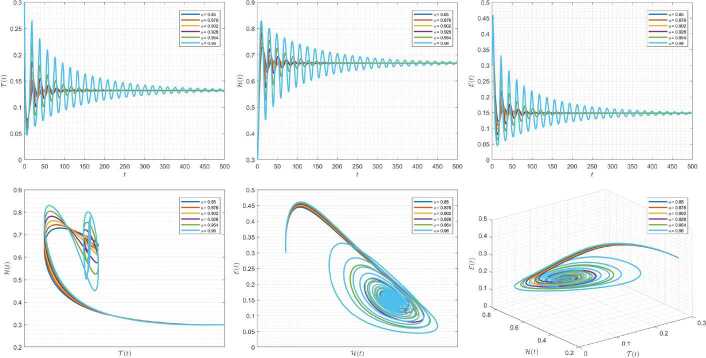
Simulations for solving (43) using (59) along with $(\mathcal{T}(0),\mathcal{H}(0),\mathcal{E}(0) )= (0.3,0.3,0.3 )$ and $\beta _{12}= 0.745$

**Figure 9:**
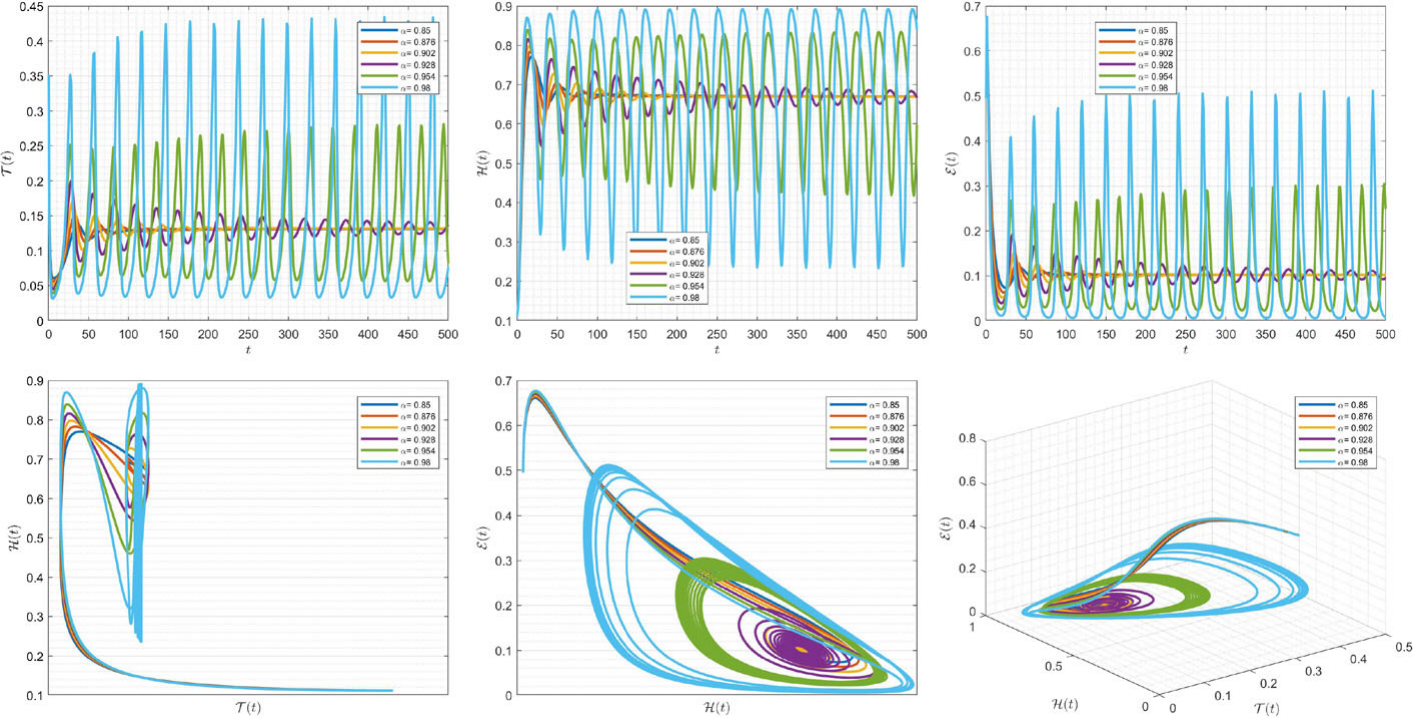
Simulations for solving (43) using (59) along with $(\mathcal{T}(0),\mathcal{H}(0),\mathcal{E}(0) )= ( 0.3517, 0.1115, 0.4951 )$ and $\beta _{12}= 0.92$

Figures 7–9 are plotted to demonstrate the variation of state variables in model (43) when the scheme (59) is used for different values of $\alpha \in (0,1]$. In this simulations, we have considered the following values in the model: $\beta _{12}=1$, $\beta _{13}=2.5$, $k_{2}=0.6$, $\beta _{21}=1.5$, $k_{3}=4.5$, $s_{3}=1$, $a_{31}=0.2$, and $d_{3}=0.5$. In our performed numerical simulations, $t_{\mathrm{final}}= 500$ and $\hbar = 0.001$. In Fig. 7, we take $(\mathcal{T}(t), \mathcal{H}(t), \mathcal{E}(t) )|_{t=0}= (0.1, 0.1, 0.1 )$. In this case the model exhibits chaotic attractor behavior. Also, taking the initial conditions $(\mathcal{T}(t), \mathcal{H}(t), \mathcal{E}(t) )|_{t=0}= (0.3, 0.3, 0.3 )$ and $\beta _{12} = 0.745 $ the model shows the limit cycle behavior in Fig. 8. Moreover, for $\mathcal{T}(0)=0.3517$, $\mathcal{H}(0)=0.1115$, $\mathcal{E}(0)=0.4951$, and $\beta _{12} = 0.920$, we get periodic orbit trajectories in the solutions as depicted in Fig. 9.

## The model via fractal-fractional derivative involving different laws

In this section, we will use several well-known fractal-fractional derivatives in model (4).

### Power-law fractal-fractional derivative

In this subsection, we replace the derivative in (4) by the fractal-fractional derivative with power-law:

60 $$ \begin{gathered} {}_{0}^{\mathsf{FF-P}} \mathcal{D}_{t}^{{\boldsymbol{\alpha }},\tau } { \mathcal{T}}(t)= \mathcal{T} (t) \bigl(1-\mathcal{T}(t) \bigr)- \beta _{12} \mathcal{T}(t)\mathcal{H}(t)- \beta _{13} \mathcal{T}(t) \mathcal{E}(t), \\ {}_{0}^{\mathsf{FF-P}} \mathcal{D}_{t}^{{\boldsymbol{\alpha }},\tau } { \mathcal{H}}(t)=k_{2} \mathcal{H} (t) \bigl(1-\mathcal{H}(t) \bigr)- \beta _{21} \mathcal{T}(t)\mathcal{H}(t), \\ {}_{0}^{\mathsf{FF-P}} \mathcal{D}_{t}^{{\boldsymbol{\alpha }},\tau } { \mathcal{E}}(t)= \frac{k_{3} \mathcal{T}(t)\mathcal{E}(t)}{\mathcal{T}(t)+s_{3}}- \beta _{31} \mathcal{T}(t) \mathcal{E}(t)-c_{3} \mathcal{E}(t), \end{gathered} $$

where the fractal-fractional derivative with power-law of function $\phi (t)$ is defined as [11]

61 $$\begin{aligned} {}^{\mathsf{FF-P}}_{0}{\mathcal{I}}_{t}^{ {\boldsymbol{\alpha }},\psi } \phi (t) = \frac{1}{\Gamma (n- {\boldsymbol{\alpha }} )}\frac{d}{d t^{\psi }} \int _{0}^{t} \frac{ \phi ({\eta } )\, d{\eta }}{(t-{\eta })^{ 1-n+{\boldsymbol{\alpha }} }}, \quad n-1< { \boldsymbol{\alpha }}, \psi \le n\in \mathbb{N}, \end{aligned}$$

and

62 $$\begin{aligned} \frac{d}{d t^{\psi }} \phi (u)=\lim_{n\rightarrow \infty } \frac{\phi (t)-\phi (u)}{t^{\psi }-u^{\psi }}. \end{aligned}$$

By employing the corresponding inverse operator in Eq. (60) and then placing $t= t_{n+1}$ in the resultant we obtain the following recursive form:

$$\begin{aligned}& \begin{aligned} \mathcal{T}_{n+1}(t) &= \mathcal{T}_{1}(0)\\ &\quad {}+ \frac{\tau }{\Gamma ({\boldsymbol{\alpha }})}\sum_{j=0}^{n} \int _{t_{j}}^{t_{j+1}}{ \eta }^{\tau -1} \bigl( \mathcal{T}({\eta }) \bigl(1-\mathcal{T}({\eta }) \bigr)-\beta _{12} \mathcal{T}({\eta })\mathcal{H}({\eta })-\beta _{13} \mathcal{T}({\eta })\mathcal{E}({\eta }) \bigr)\, d{\eta }, \end{aligned} \\& \mathcal{H}_{n+1}(t) = \mathcal{H}_{2}(0)+ \frac{\tau }{\Gamma ({\boldsymbol{\alpha }})}\sum_{j=0}^{n} \int _{t_{j}}^{t_{j+1}}{ \eta }^{\tau -1} \bigl( {\eta }_{1} \bigl[k_{2} \mathcal{H} ({\eta }) \bigl(1- \mathcal{H}({\eta }) \bigr)-\beta _{21} \mathcal{T}({\eta }) \mathcal{H}({\eta }) \bigr] \bigr)\, d{\eta }, \\& \mathcal{E}_{n+1}(t) = \mathcal{E}_{3}(0)+ \frac{\tau }{\Gamma ({\boldsymbol{\alpha }})}\sum_{\zeta =0}^{n} \int _{t_{ \zeta }}^{t_{\zeta +1}}{\eta }^{\tau -1} \biggl( \frac{k_{3} \mathcal{T}({\eta })\mathcal{E}({\eta })}{\mathcal{T}({\eta })+s_{3}}- \beta _{31} \mathcal{T}({\eta })\mathcal{E}({\eta })-c_{3} \mathcal{E}({ \eta }) \biggr)\, d{\eta }. \end{aligned}$$

Now let us define the functions

63 $$ \begin{gathered} \mathcal{K}_{1} ({\eta } )={\eta }^{\tau -1} \bigl( \mathcal{T}({\eta }) \bigl(1-\mathcal{T}({\eta }) \bigr)-\beta _{12} \mathcal{T}({\eta })\mathcal{H}({\eta })-\beta _{13} \mathcal{T}({\eta }) \mathcal{E}({\eta }) \bigr), \\ \mathcal{K}_{2} ({\eta } )={\eta }^{\tau -1} \bigl( {\eta }_{1} \bigl[k_{2} \mathcal{H} ({\eta }) \bigl(1- \mathcal{H}({\eta }) \bigr)- \beta _{21} \mathcal{T}({\eta }) \mathcal{H}({\eta }) \bigr] \bigr), \\ \mathcal{K}_{3} ({\eta } )={\eta }^{\tau -1} \biggl( \frac{k_{3} \mathcal{T}({\eta })\mathcal{E}({\eta })}{\mathcal{T}({\eta })+s_{3}}- \beta _{31} \mathcal{T}({\eta })\mathcal{E}({\eta })-c_{3} \mathcal{E}({ \eta }) \biggr). \end{gathered} $$

These functions can be interpolated in $[t_{\zeta }, t_{\zeta +1}]$ as

64 $$ \mathcal{K}_{i} ({\eta } )= \frac{{\eta }-t_{\zeta -1}}{t_{\zeta }-t_{\zeta -1}} \mathcal{K}_{i} (t_{\zeta } )-\frac{{\eta }-t_{j }}{t_{\zeta }-t_{\zeta -1}} \mathcal{K}_{i} (t_{\zeta -1} ). $$

Thus we obtain

65 $$\begin{aligned}& \mathcal{T}_{n+1} = \mathcal{T}_{0}+ \frac{\tau (\Delta t)^{{\boldsymbol{\alpha }}}}{\Gamma ({\boldsymbol{\alpha }}+2)}\sum_{ \zeta =0}^{n} \bigl[ \mathcal{K}_{1} (t_{\zeta } ) \bigl[(- \zeta +1+n)^{{\boldsymbol{\alpha }}}(- \zeta +n+{\boldsymbol{\alpha }}+2) \\& \hphantom{\mathcal{T}_{n+1} =}{}-(-\zeta +n)^{{ \boldsymbol{\alpha }}}(-\zeta +n+2{ \boldsymbol{\alpha }}+2) \bigr] \end{aligned}$$

66 $$\begin{aligned}& \hphantom{\mathcal{T}_{n+1} =}{}- \mathcal{K}_{1} (t_{\zeta -1} ) \bigl[(-\zeta +1+n)^{{ \boldsymbol{\alpha }}+1} -(-\zeta +n)^{{\boldsymbol{\alpha }}}(-\zeta +n+ { \boldsymbol{\alpha }}+1) \bigr] \bigr], \\& \mathcal{H}_{n+1} = \mathcal{H}_{0}+ \frac{\tau (\Delta t)^{{\boldsymbol{\alpha }}}}{\Gamma ({\boldsymbol{\alpha }}+2)}\sum _{ \zeta =0}^{n} \bigl[ \mathcal{K}_{2} (t_{\zeta } ) \bigl[(- \zeta +1+n)^{{\boldsymbol{\alpha }}}(-\zeta +n+{\boldsymbol{ \alpha }}+2) \\& \hphantom{\mathcal{H}_{n+1} =}{}-(-\zeta +n)^{{ \boldsymbol{\alpha }}}(-\zeta +n+2{\boldsymbol{\alpha }}+2) \bigr] \end{aligned}$$

67 $$\begin{aligned}& \hphantom{\mathcal{H}_{n+1} =}{}-\mathcal{K}_{2} (t_{\zeta -1} ) \bigl[(-\zeta +1+n)^{{\boldsymbol{\alpha }}+1} -(-\zeta +n)^{{\boldsymbol{\alpha }}}(-\zeta +n+ {\boldsymbol{\alpha }}+1) \bigr] \bigr], \end{aligned}$$

68 $$\begin{aligned}& \mathcal{E}_{n+1} = \mathcal{E}_{0}+ \frac{\tau (\Delta t)^{{\boldsymbol{\alpha }}}}{\Gamma ({\boldsymbol{\alpha }}+2)}\sum _{ \zeta =0}^{n} \bigl[ \mathcal{K}_{3} (t_{\zeta } ) \bigl[(- \zeta +1+n)^{{\boldsymbol{\alpha }}}(-\zeta +n+{\boldsymbol{ \alpha }}+2) \\& \hphantom{\mathcal{E}_{n+1} =}{}-(-\zeta +n)^{{ \boldsymbol{\alpha }}}(-\zeta +n+2{\boldsymbol{\alpha }}+2) \bigr] \\& \hphantom{\mathcal{E}_{n+1} =}{}-\mathcal{K}_{3} (t_{\zeta -1} ) \bigl[(-\zeta +1+n)^{{\boldsymbol{\alpha }}+1} -(-\zeta +n)^{{\boldsymbol{\alpha }}}(-\zeta +n+ { \boldsymbol{\alpha }}+1) \bigr] \bigr], \end{aligned}$$

where $\mathcal{K}_{j}$, $\zeta =1, 2, 3$, are given in (63).

### Numerical simulations

**Figure 10:**
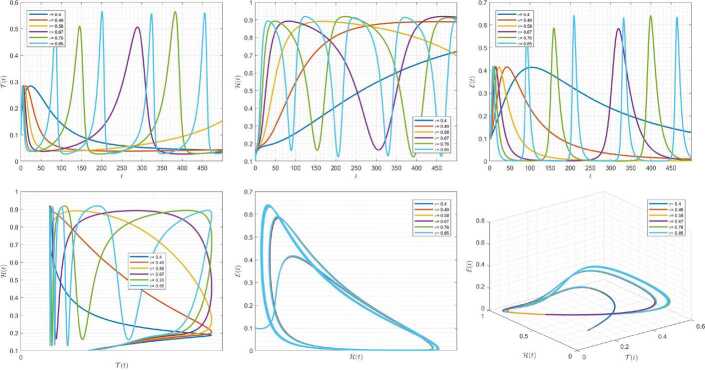
Simulations for solving (60) using (65) along with $(\mathcal{T}(0),\mathcal{H}(0),\mathcal{E}(0) )= (0.1,0.1,0.1 )$

**Figure 11:**
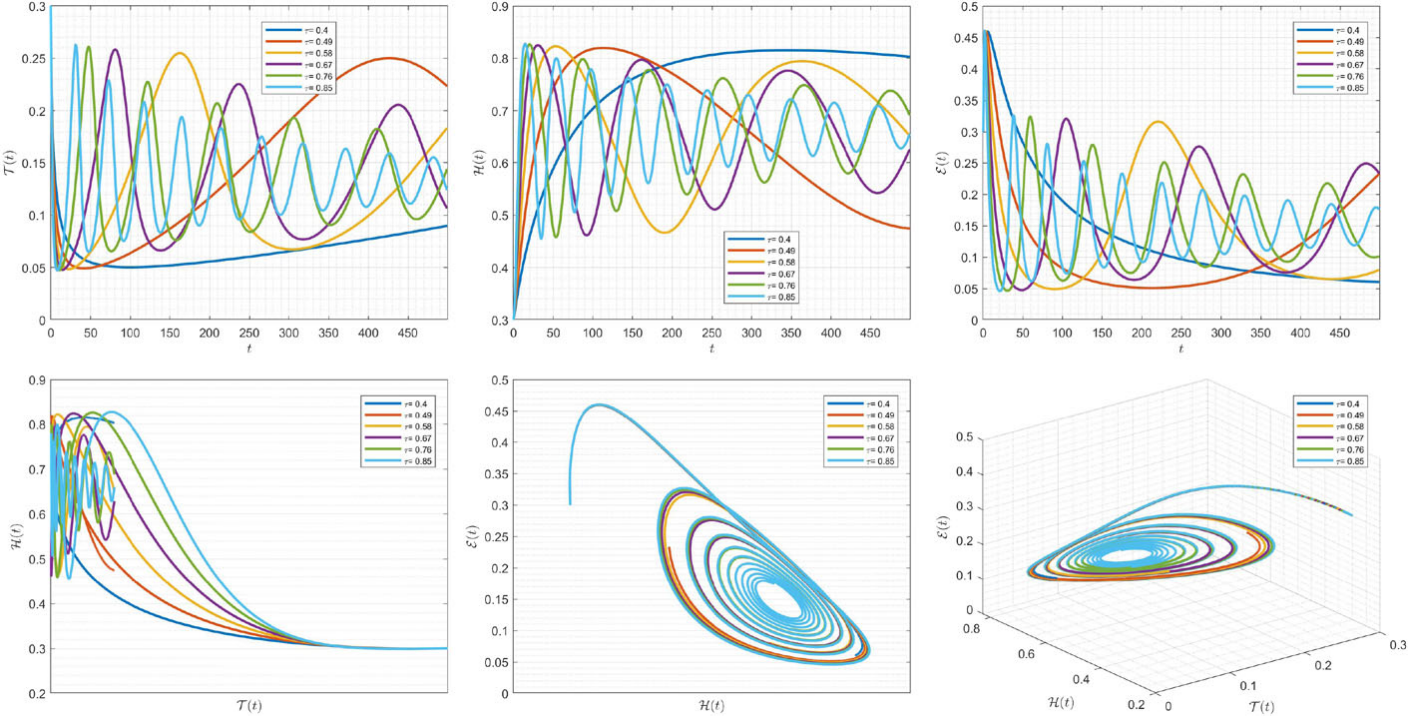
Simulations for solving (60) using (65) along with $(\mathcal{T}(0),\mathcal{H}(0),\mathcal{E}(0) )= (0.3,0.3,0.3 )$ and $\beta _{12}= 0.745$

**Figure 12:**
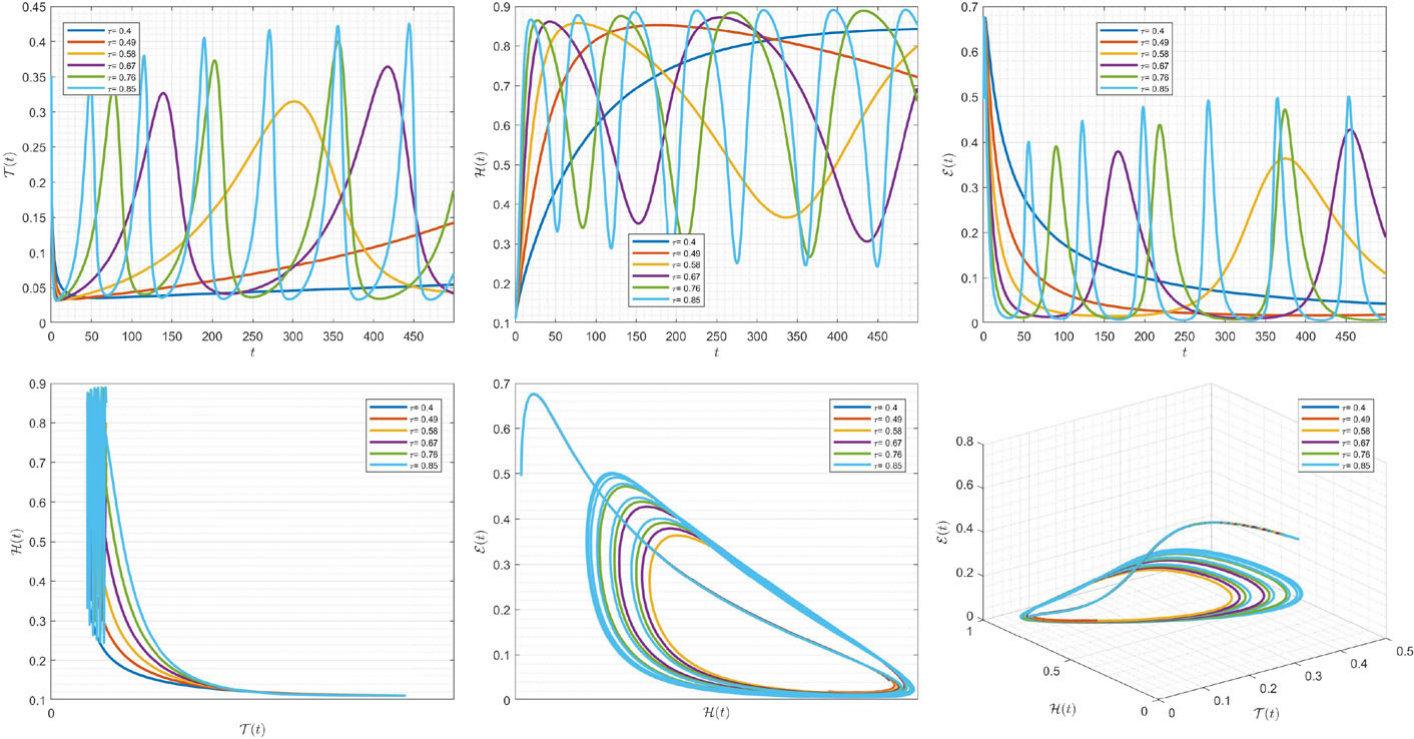
Simulations for solving (60) using (65) along with $(\mathcal{T}(0),\mathcal{H}(0),\mathcal{E}(0) )= ( 0.3517, 0.1115, 0.4951 )$ and $\beta _{12}= 0.92$

Figures 10–12 demonstrate the variation of state variables in model (60) while applying scheme (65) for different values of $\tau \in (0,1]$, and $\alpha =0.95$. In this simulations, we considered the following values in the model: $\beta _{12}=1$, $\beta _{13}=2.5$, $k_{2}=0.6$, $\beta _{21}=1.5$, $k_{3}=4.5$, $s_{3}=1$, $a_{31}=0.2$, and $d_{3}=0.5$. In our numerical simulations, $t_{\mathrm{final}}= 500$ and $\hbar = 0.001$. In Fig. 10, we put $(\mathcal{T}(t), \mathcal{H}(t), \mathcal{E}(t) )|_{t=0}= (0.1, 0.1, 0.1 )$. In this case the model exhibits chaotic attractor behavior. Also, by imposing the initial conditions $(\mathcal{T}(t), \mathcal{H}(t), \mathcal{E}(t) )|_{t=0}= (0.3, 0.3, 0.3 )$ and $\beta _{12} = 0.745 $ the model shows the limit cycle behavior in Fig. 11. Further, for $\mathcal{T}(0)=0.3517$, $\mathcal{H}(0)=0.1115$, $\mathcal{E}(0)=0.4951$, and $\beta _{12} = 0.920$, Fig. 12 confirms the periodic orbit trajectories in the solutions.

### Exponential decay-law fractal-fractional derivative

In this part, we replace the derivative in (4) by the fractal-fractional derivative with exponential decay-law:

69 $$ \begin{gathered} {}_{0}^{\mathsf{FF-E}} \mathcal{D}_{t}^{{\boldsymbol{\alpha }},\tau } { \mathcal{T}}(t)= \mathcal{T} (t) \bigl(1-\mathcal{T}(t) \bigr)- \beta _{12} \mathcal{T}(t)\mathcal{H}(t)- \beta _{13} \mathcal{T}(t) \mathcal{E}(t), \\ {}_{0}^{\mathsf{FF-E}} \mathcal{D}_{t}^{{\boldsymbol{\alpha }},\tau } { \mathcal{H}}(t)=k_{2} \mathcal{H} (t) \bigl(1-\mathcal{H}(t) \bigr)- \beta _{21} \mathcal{T}(t)\mathcal{H}(t), \\ {}_{0}^{\mathsf{FF-E}} \mathcal{D}_{t}^{{\boldsymbol{\alpha }},\tau } { \mathcal{E}}(t)= \frac{k_{3} \mathcal{T}(t)\mathcal{E}(t)}{\mathcal{T}(t)+s_{3}}- \beta _{31} \mathcal{T}(t) \mathcal{E}(t)-c_{3} \mathcal{E}(t), \end{gathered} $$

where the fractal-fractional derivative with exponential decay law of a function ${\phi }(t)$ is defined as [11]

70 $$ {}^{\mathsf{FF-E}}_{0}{\mathcal{I}}_{t}^{ {\boldsymbol{\alpha }},\psi } {\phi }(t) = \frac{\mathsf{M}({\boldsymbol{\alpha }})}{ n-{{{\boldsymbol{\alpha }}}}} \frac{d}{d t^{\psi }} \int _{0}^{t} {\phi }({\eta } )\exp \biggl[- \frac{{\boldsymbol{\alpha }}}{n-{\boldsymbol{\alpha }}}(t-{\eta }) \biggr] d{\eta }, \quad n-1< {\boldsymbol{\alpha }}, \psi \le n\in \mathbb{N}, $$

and $\frac{d}{d t^{\psi }} \phi (u)$ is introduced in (62).

Applying the Caputo–Fabrizio integral to Eq. (69), we obtain

71 $$ \begin{gathered} {\mathcal{T}}(t)= {\mathcal{T}}(0) + \frac{\gamma _{1}}{\mathsf{M}({\boldsymbol{\alpha }})} \mathcal{F}_{1} \bigl(t , \mathcal{T}(t), \mathcal{H}(t), \mathcal{E}(t), x_{4}(t) \bigr)\\ \hphantom{{\mathcal{T}}(t)=}{}+ \frac{\gamma _{1}{\boldsymbol{\alpha }}}{\mathsf{M}({\boldsymbol{\alpha }})} \int _{ 0}^{t} \mathcal{F}_{1} \bigl({ \eta },\mathcal{T}({\eta }),\mathcal{H}({\eta }), \mathcal{E}({\eta }) \bigr)\, d{ \eta }, \\ {\mathcal{H}}(t)= {\mathcal{H}}(0) + \frac{\gamma _{2}}{\mathsf{M}({\boldsymbol{\alpha }})} \mathcal{F}_{2} \bigl(t , \mathcal{T}(t), \mathcal{H}(t), \mathcal{E}(t), x_{4}(t) \bigr)\\ \hphantom{{\mathcal{H}}(t)=}{}+ \frac{\gamma _{2}{\boldsymbol{\alpha }}}{\mathsf{M}({\boldsymbol{\alpha }})} \int _{ 0}^{t} \mathcal{F}_{2} \bigl({\eta },\mathcal{T}({\eta }),\mathcal{H}({\eta }), \mathcal{E}({\eta }) \bigr)\, d{\eta }, \\ {\mathcal{E}}(t)= {\mathcal{E}}(0) + \frac{\gamma _{3}}{\mathsf{M}({\boldsymbol{\alpha }})} \mathcal{F}_{3} \bigl(t , \mathcal{T}(t), \mathcal{H}(t), \mathcal{E}(t), x_{4}(t) \bigr)\\ \hphantom{{\mathcal{E}}(t)=}{}+ \frac{\gamma _{3}{\boldsymbol{\alpha }}}{\mathsf{M}({\boldsymbol{\alpha }})} \int _{ 0}^{t} \mathcal{F}_{3} \bigl({ \eta },\mathcal{T}({\eta }),\mathcal{H}({\eta }), \mathcal{E}({\eta }) \bigr)\, d{ \eta }. \end{gathered} $$

Setting $t=t_{n+1}$ in (71), based on the proposed scheme in [11], we get

72 $$ \begin{gathered} {\mathcal{T}_{n+1}} = { \mathcal{T}}(0) + \frac{\tau }{\mathsf{M}({\boldsymbol{\alpha }})} \mathcal{F}_{1} (t_{n}, \mathcal{T}_{n} , \mathcal{H}_{n} , \mathcal{E}_{n} )+ \frac{\tau {\boldsymbol{\alpha }}}{\mathsf{M}({\boldsymbol{\alpha }})} \int _{ 0}^{t_{n+1}} \mathcal{F}_{1} \bigl({ \eta },\mathcal{T}({\eta }),\mathcal{H}({\eta }), \mathcal{E}({\eta }) \bigr)\, d{ \eta }, \\ {\mathcal{H}_{n+1}} = {\mathcal{H}}(0) + \frac{\tau }{\mathsf{M}({\boldsymbol{\alpha }})} \mathcal{F}_{2} (t_{n }, \mathcal{T}_{n} , \mathcal{H}_{n} , \mathcal{E}_{n} )+ \frac{\tau {\boldsymbol{\alpha }}}{\mathsf{M}({\boldsymbol{\alpha }})} \int _{ 0}^{t_{n+1}} \mathcal{F}_{2} \bigl({ \eta },\mathcal{T}({\eta }),\mathcal{H}({\eta }), \mathcal{E}({\eta }) \bigr)\, d{ \eta }, \\ {\mathcal{E}_{n+1}} = {\mathcal{E}}(0) + \frac{\tau }{\mathsf{M}({\boldsymbol{\alpha }})} \mathcal{F}_{3} (t_{n }, \mathcal{T}_{n} , \mathcal{H}_{n} , \mathcal{E}_{n} )+ \frac{\tau {\boldsymbol{\alpha }}}{\mathsf{M}({\boldsymbol{\alpha }})} \int _{ 0}^{t_{n+1}} \mathcal{F}_{3} \bigl({ \eta },\mathcal{T}({\eta }),\mathcal{H}({\eta }), \mathcal{E}({\eta }) \bigr)\, d{ \eta }. \end{gathered} $$

Taking the difference between ${\mathcal{T}_{n+1}} $ and ${\mathcal{T}_{n }} $ yields

73 $$ \begin{gathered} {\mathcal{T}_{n+1}} - { \mathcal{T}_{n}} = \frac{\tau }{\mathsf{M}({\boldsymbol{\alpha }})} \bigl[\mathcal{F}_{1} (t_{n}, \mathcal{T}_{n} , \mathcal{H}_{n} , \mathcal{E}_{n} )- \mathcal{F}_{1} (t_{n-1}, \mathcal{T}_{n-1} , \mathcal{H}_{n-1} , \mathcal{E}_{n-1} ) \bigr] \\ \hphantom{{\mathcal{T}_{n+1}} - { \mathcal{T}_{n}} =}{}+\frac{\tau {\boldsymbol{\alpha }}}{\mathsf{M}({\boldsymbol{\alpha }})} \int _{ t_{n}}^{t_{n+1}} \mathcal{F}_{1} \bigl({ \eta },\mathcal{T}({\eta }),\mathcal{H}({\eta }), \mathcal{E}({\eta }) \bigr)\, d{ \eta }, \\ {\mathcal{H}_{n+1}} - {\mathcal{H}_{n}} = \frac{\tau }{\mathsf{M}({\boldsymbol{\alpha }})} \bigl[\mathcal{F}_{2} (t_{n}, \mathcal{T}_{n} , \mathcal{H}_{n} , \mathcal{E}_{n} )- \mathcal{F}_{2} (t_{n-1}, \mathcal{T}_{n-1} , \mathcal{H}_{n-1} , \mathcal{E}_{n-1} ) \bigr] \\ \hphantom{{\mathcal{H}_{n+1}} - {\mathcal{H}_{n}} =}{}+\frac{\tau {\boldsymbol{\alpha }}}{\mathsf{M}({\boldsymbol{\alpha }})} \int _{ t_{n}}^{t_{n+1}} \mathcal{F}_{2} \bigl({ \eta },\mathcal{T}({\eta }),\mathcal{H}({\eta }), \mathcal{E}({\eta }) \bigr)\, d{ \eta }, \\ {\mathcal{E}_{n+1}} - {\mathcal{E}_{n}} = \frac{\tau }{\mathsf{M}({\boldsymbol{\alpha }})} \bigl[\mathcal{F}_{3} (t_{n}, \mathcal{T}_{n} , \mathcal{H}_{n} , \mathcal{E}_{n} )- \mathcal{F}_{3} (t_{n-1}, \mathcal{T}_{n-1} , \mathcal{H}_{n-1} , \mathcal{E}_{n-1} ) \bigr] \\ \hphantom{{\mathcal{E}_{n+1}} - {\mathcal{E}_{n}} =}{}+\frac{\tau {\boldsymbol{\alpha }}}{\mathsf{M}({\boldsymbol{\alpha }})} \int _{ t_{n}}^{t_{n+1}} \mathcal{F}_{3} \bigl({ \eta },\mathcal{T}({\eta }),\mathcal{H}({\eta }), \mathcal{E}({\eta }) \bigr)\, d{ \eta }. \end{gathered} $$

Therefore the approximate solution to the problem can be determined using the following iterative procedures:

74 $$ \begin{gathered} {\mathcal{T}_{n+1}} = { \mathcal{T}_{n}} + \frac{\tau }{\mathsf{M}({\boldsymbol{\alpha }})} \bigl[\mathcal{F}_{1} (t_{n}, \mathcal{T}_{n} , \mathcal{H}_{n} , \mathcal{E}_{n} )- \mathcal{F}_{1} (t_{n-1}, \mathcal{T}_{n-1} , \mathcal{H}_{n-1} , \mathcal{E}_{n-1} ) \bigr] \\ \hphantom{{\mathcal{T}_{n+1}} =}{}+\frac{\tau {\boldsymbol{\alpha }}}{\mathsf{M}({\boldsymbol{\alpha }})} \biggl[ \frac{3\Delta }{2} \mathcal{F}_{1} (t_{n}, \mathcal{T}_{n} , \mathcal{H}_{n} , \mathcal{E}_{n} )-\frac{\Delta }{2} \mathcal{F}_{1} (t_{n-1}, x_{n-1} , \mathcal{H}_{n-1} , \mathcal{E}_{n-1} ) \biggr], \\ {\mathcal{H}_{n+1}} = {\mathcal{H}_{n}} + \frac{\tau }{\mathsf{M}({\boldsymbol{\alpha }})} \bigl[\mathcal{F}_{2} (t_{n}, \mathcal{T}_{n} , \mathcal{H}_{n} , \mathcal{E}_{n} )- \mathcal{F}_{2} (t_{n-1}, \mathcal{T}_{n-1} , \mathcal{H}_{n-1} , \mathcal{E}_{n-1} ) \bigr] \\ \hphantom{{\mathcal{H}_{n+1}} =}{}+\frac{\tau {\boldsymbol{\alpha }}}{\mathsf{M}({\boldsymbol{\alpha }})} \biggl[ \frac{3\Delta }{2} \mathcal{F}_{2} (t_{n}, \mathcal{T}_{n} , \mathcal{H}_{n} , \mathcal{E}_{n} )-\frac{\Delta }{2} \mathcal{F}_{2} (t_{n-1}, \mathcal{T}_{n-1} , y_{n-1} , \mathcal{E}_{n-1} ) \biggr], \\ {\mathcal{E}_{n+1}} = {\mathcal{E}_{n}} + \frac{\tau }{\mathsf{M}({\boldsymbol{\alpha }})} \bigl[\mathcal{F}_{3} (t_{n}, \mathcal{T}_{n} , \mathcal{H}_{n} , \mathcal{E}_{n} )- \mathcal{F}_{3} (t_{n-1}, \mathcal{T}_{n-1} , \mathcal{H}_{n-1} , \mathcal{E}_{n-1} ) \bigr] \\ \hphantom{{\mathcal{E}_{n+1}} =}{}+\frac{\tau {\boldsymbol{\alpha }}}{\mathsf{M}({\boldsymbol{\alpha }})} \biggl[ \frac{3\Delta }{2} \mathcal{F}_{3} (t_{n}, \mathcal{T}_{n} , \mathcal{H}_{n} , \mathcal{E}_{n} )-\frac{\Delta }{2} \mathcal{F}_{3} (t_{n-1}, \mathcal{T}_{n-1}, \mathcal{H}_{n-1} , \mathcal{E}_{n-1} ) \biggr], \end{gathered} $$

where $\mathcal{F}_{j}$, $\zeta =1, 2, 3$, are given in (63).

### Numerical simulations

**Figure 13:**
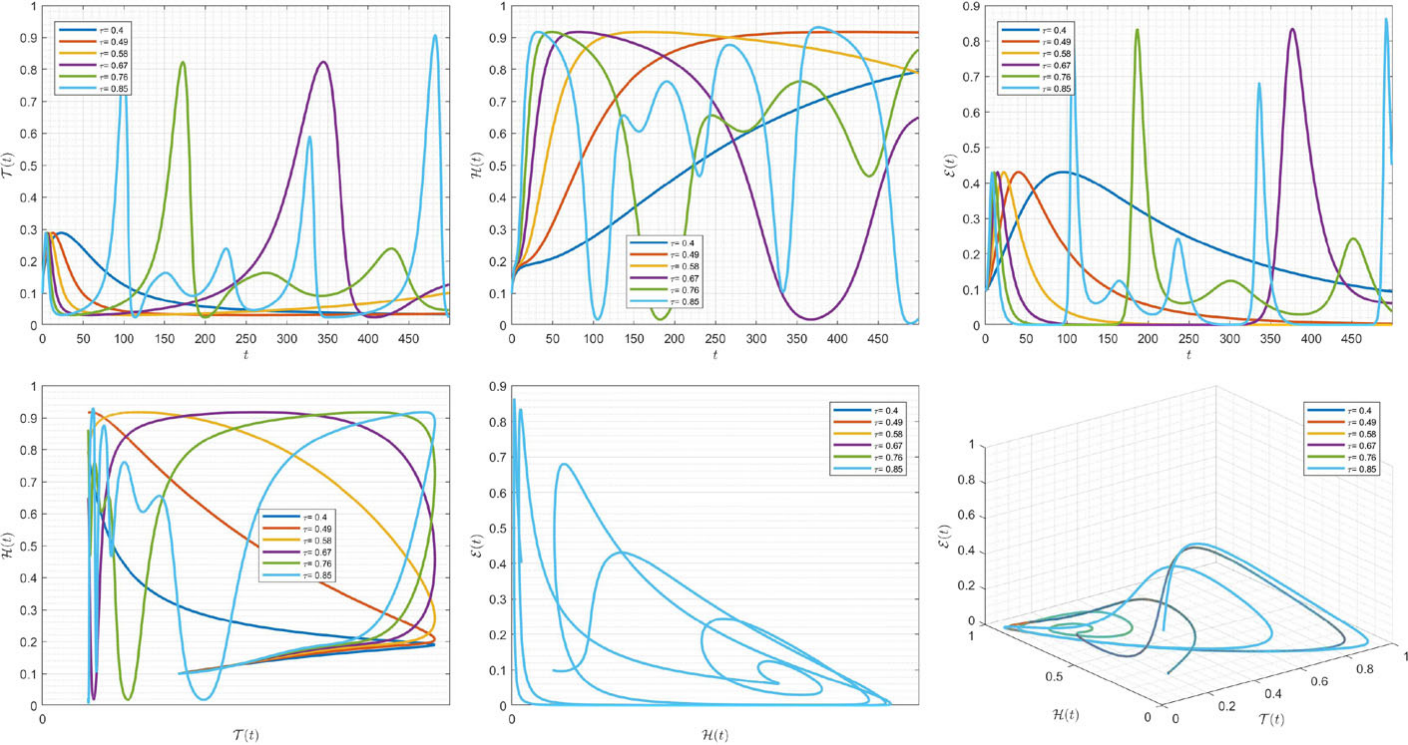
Simulations for solving (69) using (74) along with $(\mathcal{T}(0),\mathcal{H}(0),\mathcal{E}(0) )= (0.1,0.1,0.1 )$

**Figure 14:**
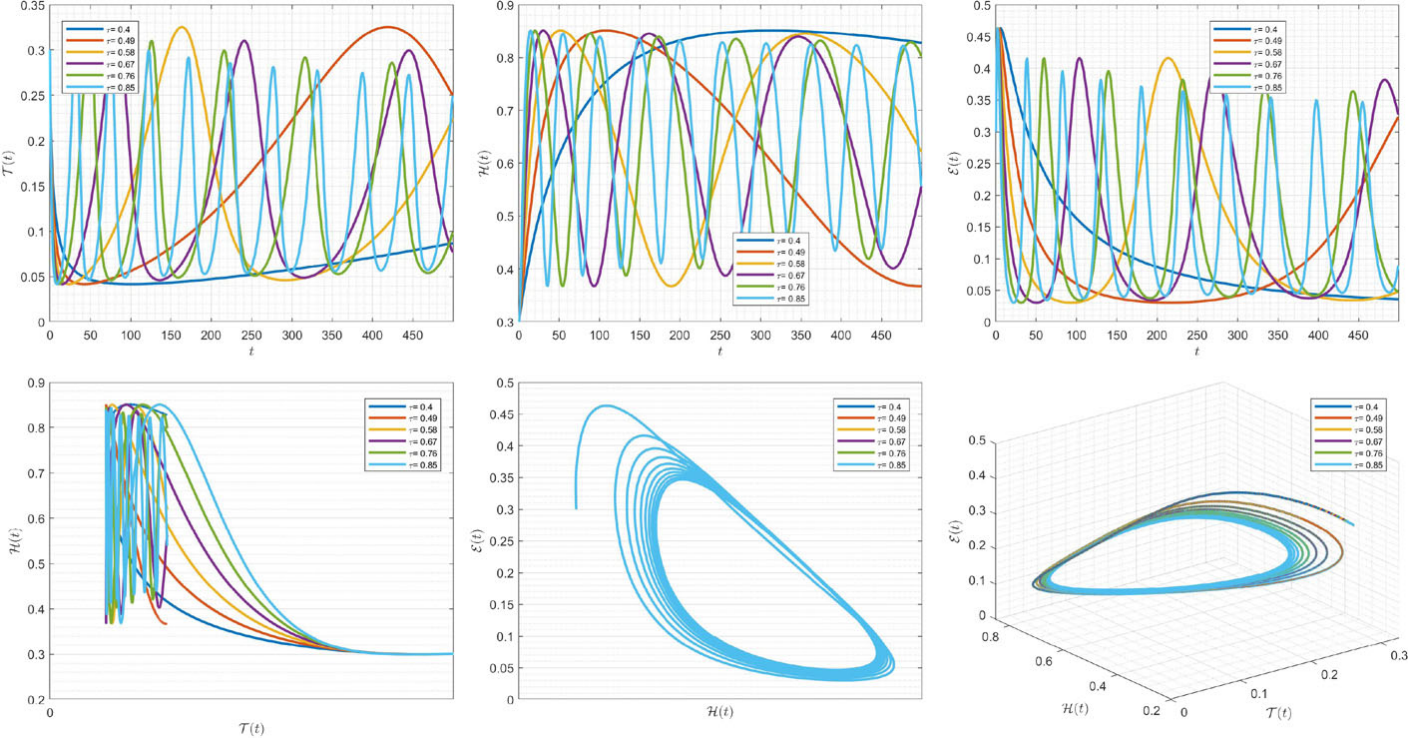
Simulations for solving (69) using (74) along with $(\mathcal{T}(0),\mathcal{H}(0),\mathcal{E}(0) )= (0.3,0.3,0.3 )$ and $\beta _{12}= 0.745$

**Figure 15:**
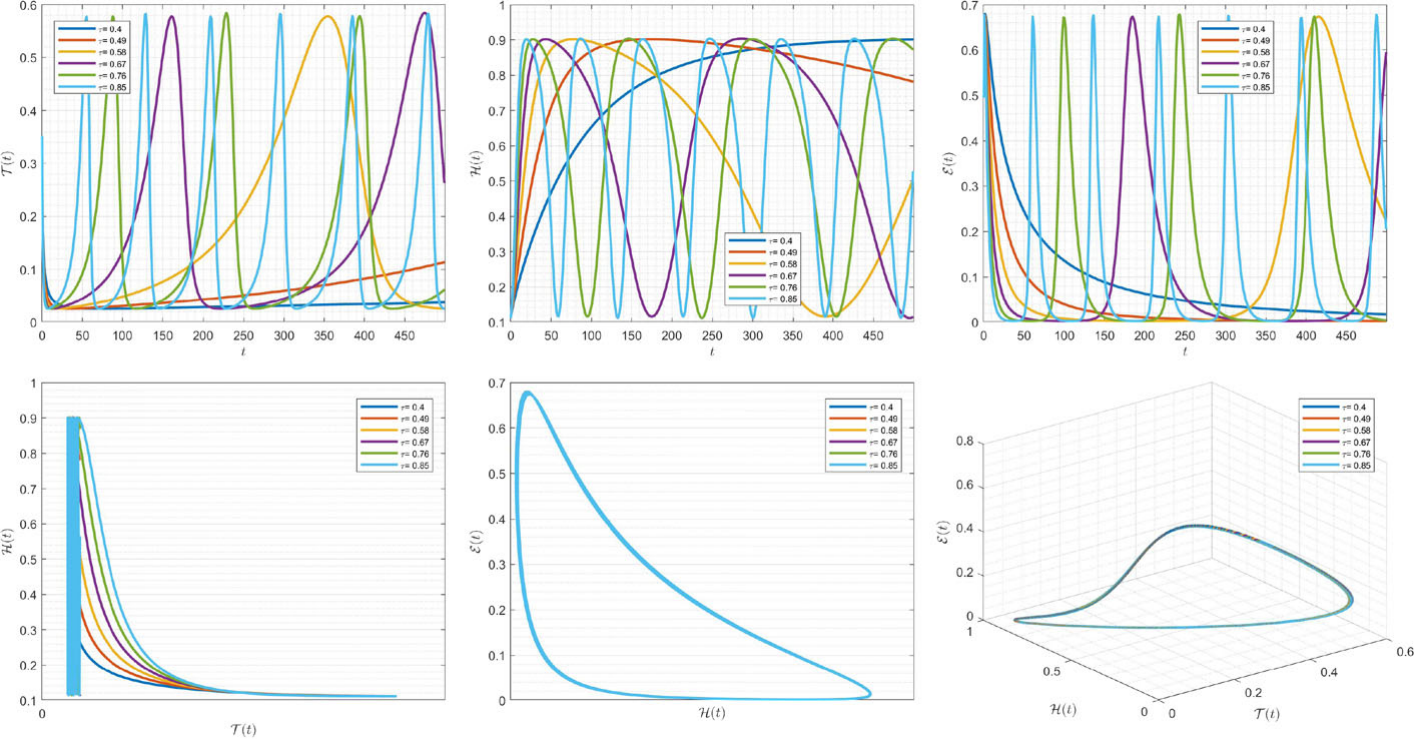
Simulations for solving (69) using (74) along with $(\mathcal{T}(0),\mathcal{H}(0),\mathcal{E}(0) )= ( 0.3517, 0.1115, 0.4951 )$ and $\beta _{12}= 0.92$

Figures 13–15 demonstrate the variation of state variables in model (69) when scheme (74) is applied for different values of $\tau \in (0,1]$ and $\alpha =0.95$. In this simulations, we considered the following values in the model: $\beta _{12}=1$, $\beta _{13}=2.5$, $k_{2}=0.6$, $\beta _{21}=1.5$, $k_{3}=4.5$, $s_{3}=1$, $a_{31}=0.2$, and $d_{3}=0.5$. In our numerical simulations, $t_{\mathrm{final}}= 500$ and $\hbar = 0.001$. In Fig. 13, we take $(\mathcal{T}(t), \mathcal{H}(t), \mathcal{E}(t) )|_{t=0}= (0.1, 0.1, 0.1 )$. In this case the model exhibits chaotic attractor behavior. Further, for $(\mathcal{T}(t), \mathcal{H}(t), \mathcal{E}(t) )|_{t=0}= (0.3, 0.3, 0.3 )$ and $\beta _{12} = 0.745 $, the model shows the limit cycle behavior in Fig. 14. Also, letting $\mathcal{T}(0)=0.3517$, $\mathcal{H}(0)=0.1115$, $\mathcal{E}(0)=0.4951$, and $\beta _{12} = 0.920$, Fig. 15 confirms the periodic orbit trajectories in the solutions.

### Mittag-Leffler-law fractal-fractional derivative

In this subsection, we use the fractal-fractional derivative with Mittag-Leffler law in (4). So, we achieve

75 $$ \begin{gathered} {}_{0}^{\mathsf{FF-ABC}} \mathcal{D}_{t}^{{\boldsymbol{\alpha }},\tau } { \mathcal{T}}(t)= \mathcal{T} (t) \bigl(1-\mathcal{T}(t) \bigr)- \beta _{12} \mathcal{T}(t)\mathcal{H}(t)- \beta _{13} \mathcal{T}(t) \mathcal{E}(t), \\ {}_{0}^{\mathsf{FF-ABC}} \mathcal{D}_{t}^{{\boldsymbol{\alpha }},\tau } { \mathcal{H}}(t)=k_{2} \mathcal{H} (t) \bigl(1-\mathcal{H}(t) \bigr)- \beta _{21} \mathcal{T}(t)\mathcal{H}(t), \\ {}_{0}^{\mathsf{FF-ABC}} \mathcal{D}_{t}^{{\boldsymbol{\alpha }},\tau } { \mathcal{E}}(t)= \frac{k_{3} \mathcal{T}(t)\mathcal{E}(t)}{\mathcal{T}(t)+s_{3}}- \beta _{31} \mathcal{T}(t) \mathcal{E}(t)-c_{3} \mathcal{E}(t), \end{gathered} $$

where the fractal-fractional derivative with Mittag-Leffler law of a unction ${\phi }(t)$ is defined as [11]

76 $$\begin{aligned} & {0}^{\mathsf{FF-ABC}} \mathcal{D}_{t}^{{\boldsymbol{\alpha }},\tau } {\phi }(t) \\ &\quad = \frac{\mathsf{B}({\boldsymbol{\alpha }})}{ n-{{{\boldsymbol{\alpha }}}}} \frac{d}{d t^{\psi }} \int _{0}^{t} {\phi }({\eta } )E_{\boldsymbol{\alpha }} \biggl[- \frac{{\boldsymbol{\alpha }}}{n-{\boldsymbol{\alpha }}}(t-{\eta })^{\boldsymbol{\alpha }} \biggr] d{ \eta }, \quad n-1< {\boldsymbol{\alpha }}, \psi \le n\in \mathbb{N}, \end{aligned}$$

and $\frac{d}{d t^{\psi }} \phi (u)$ is introduced in (62).

Applying the Atangana–Baleanu integral to (75), we obtain

77 $$ \begin{gathered} {\mathcal{T}}(t)= {\mathcal{T}}(0) + \frac{\tau t^{\tau -1} (1-{\boldsymbol{\alpha }})}{\Gamma ({\boldsymbol{\alpha }})} \mathcal{F}_{1} \bigl(t , \mathcal{T}(t), \mathcal{H}(t), \mathcal{E}(t), x_{4}(t) \bigr)\\ \hphantom{{\mathcal{T}}(t)=}{}+ \frac{\tau {\boldsymbol{\alpha }}}{\Gamma ({\boldsymbol{\alpha }})\mathsf{B}({\boldsymbol{\alpha }})} \int _{ 0}^{t}\delta ^{\tau -1} \mathcal{F}_{1} \bigl({\eta }, \mathcal{T}({\eta }),\mathcal{H}({\eta }),\mathcal{E}({\eta }) \bigr) (t-{ \eta })^{{\boldsymbol{\alpha }}-1}\, d{\eta }, \\ {\mathcal{H}}(t)= {\mathcal{H}}(0) + \frac{\tau t^{\tau -1}(1-{\boldsymbol{\alpha }})}{\Gamma ({\boldsymbol{\alpha }})} \mathcal{F}_{2} \bigl(t , \mathcal{T}(t), \mathcal{H}(t), \mathcal{E}(t), x_{4}(t) \bigr)\\ \hphantom{{\mathcal{H}}(t)=}{}+ \frac{\tau {\boldsymbol{\alpha }}}{\Gamma ({\boldsymbol{\alpha }})\mathsf{B}({\boldsymbol{\alpha }})} \int _{ 0}^{t}\delta ^{\tau -1} \mathcal{F}_{2} \bigl({\eta }, \mathcal{T}({\eta }),\mathcal{H}({\eta }),\mathcal{E}({\eta }) \bigr) (t-{ \eta })^{{\boldsymbol{\alpha }}-1}\, d{\eta }, \\ {\mathcal{E}}(t)= {\mathcal{E}}(0) + \frac{\tau t^{\tau -1}(1-{\boldsymbol{\alpha }})}{\Gamma ({\boldsymbol{\alpha }})} \mathcal{F}_{3} \bigl(t , \mathcal{T}(t), \mathcal{H}(t), \mathcal{E}(t), x_{4}(t) \bigr)\\ \hphantom{{\mathcal{E}}(t)=}{}+ \frac{\tau {\boldsymbol{\alpha }}}{\Gamma ({\boldsymbol{\alpha }})\mathsf{B}({\boldsymbol{\alpha }})} \int _{ 0}^{t}\delta ^{\tau -1} \mathcal{F}_{3} \bigl({\eta }, \mathcal{T}({\eta }),\mathcal{H}({\eta }),\mathcal{E}({\eta }) \bigr) (t-{ \eta })^{{\boldsymbol{\alpha }}-1}\, d{\eta }. \end{gathered} $$

Based on the scheme proposed in [11], we get

78 $$\begin{aligned}& \begin{aligned} {\mathcal{T}_{n+1}}& = { \mathcal{T}_{0}} + \frac{\tau {t_{n}^{\alpha -1}}(1-{\boldsymbol{\alpha }})}{\Gamma ({\boldsymbol{\alpha }})} \mathcal{F}_{1} (t_{n}, \mathcal{T}_{n} , \mathcal{H}_{n} , \mathcal{E}_{n} ) \\ &\quad {}+ \frac{\tau {\boldsymbol{\alpha }}}{\Gamma ({\boldsymbol{\alpha }})\mathsf{B}({\boldsymbol{\alpha }})} \sum_{\zeta =0}^{n} \int _{t_{\zeta }}^{t_{j+1}}\delta ^{\tau -1} \mathcal{F}_{1} \bigl({\eta },\mathcal{T}({\eta }),\mathcal{H}({\eta }), \mathcal{E}({\eta }) \bigr) (t_{n+1}-{\eta })^{{\boldsymbol{\alpha }}-1}\, d{\eta }, \end{aligned} \\& \begin{aligned} {\mathcal{H}_{n+1}} &= {\mathcal{H}_{0}}+ \frac{\tau {t_{n}^{\alpha -1}}(1-{\boldsymbol{\alpha }})}{\Gamma ({\boldsymbol{\alpha }})} \mathcal{F}_{2} (t_{n }, \mathcal{T}_{n} , \mathcal{H}_{n} , \mathcal{E}_{n} ) \\ &\quad {}+ \frac{\tau {\boldsymbol{\alpha }}}{\Gamma ({\boldsymbol{\alpha }})\mathsf{B}({\boldsymbol{\alpha }})} \sum_{\zeta =0}^{n} \int _{t_{\zeta }}^{t_{j+1}}\delta ^{\tau -1} \mathcal{F}_{2} \bigl({\eta },\mathcal{T}({\eta }),\mathcal{H}({\eta }), \mathcal{E}({\eta }) \bigr) (t_{n+1}-{\eta })^{{\boldsymbol{\alpha }}-1}\, d{\eta }, \end{aligned} \\& \begin{aligned} {\mathcal{E}_{n+1}} &= {\mathcal{E}_{0}} + \frac{\tau {t_{n}^{\alpha -1}}(1-{\boldsymbol{\alpha }})}{\Gamma ({\boldsymbol{\alpha }})} \mathcal{F}_{3} (t_{n }, \mathcal{T}_{n} , \mathcal{H}_{n} , \mathcal{E}_{n} ) \\ &\quad {}+ \frac{\tau {\boldsymbol{\alpha }}}{\Gamma ({\boldsymbol{\alpha }})\mathsf{B}({\boldsymbol{\alpha }})} \sum_{\zeta =0}^{n} \int _{t_{\zeta }}^{t_{j+1}}\delta ^{\tau -1} \mathcal{F}_{3} \bigl({\eta },\mathcal{T}({\eta }),\mathcal{H}({\eta }), \mathcal{E}({\eta }) \bigr) (t_{n+1}-{\eta })^{{\boldsymbol{\alpha }}-1}\, d{\eta }. \end{aligned} \end{aligned}$$

Now using the Lagrange polynomial piecewise interpolation given by Eq. (64), we obtain

79 $$\begin{aligned}& \mathcal{T}_{n+1} = \mathcal{T}_{0}+ \frac{\tau {t_{n}^{\alpha -1}}(1-{\boldsymbol{\alpha }})}{\Gamma ({{{\boldsymbol{\alpha }}}})} \mathcal{F}_{1} (t_{n}, \mathcal{T}_{n} , \mathcal{H}_{n} , \mathcal{E}_{n} ) \\& \hphantom{\mathcal{T}_{n+1} =}{}+ \frac{\tau \hbar ^{{\boldsymbol{\alpha }}}}{\Gamma ({\boldsymbol{\alpha }}+2)\mathsf{B}({\boldsymbol{\alpha }})} \sum_{\zeta =0}^{n} \bigl[ {t_{\zeta }^{\alpha -1}}\mathcal{F}_{1} (t_{\zeta },\mathcal{T}_{\zeta },\mathcal{H}_{\zeta }, \mathcal{E} _{ \zeta } ) \bigl[(-\zeta +1+n)^{{\boldsymbol{\alpha }}}(-\zeta +n+{ \boldsymbol{\alpha }}+2) \\& \hphantom{\mathcal{T}_{n+1} =}{}-(-\zeta +n)^{{\boldsymbol{\alpha }}}(-\zeta +n+2{\boldsymbol{\alpha }}+2) \bigr] \\& \hphantom{\mathcal{T}_{n+1} =}{}- {t_{\zeta -1}^{\alpha -1}}\mathcal{F}_{1} (t_{\zeta -1}, \mathcal{T}_{\zeta -1},\mathcal{H}_{\zeta -1}, \mathcal{E} _{\zeta -1} ) \bigl[(-\zeta +1+n)^{{\boldsymbol{\alpha }}+1} -(-\zeta +n)^{{\boldsymbol{\alpha }}}(-\zeta +n+ {\boldsymbol{\alpha }}+1) \bigr] \bigr], \\& \begin{gathered} \mathcal{H}_{n+1} = \mathcal{H}_{0}+ \frac{\tau {t_{n}^{\alpha -1}}(1-{\boldsymbol{\alpha }})}{\Gamma ({\boldsymbol{\alpha }})} \mathcal{F}_{2} (t_{n }, \mathcal{T}_{n} , \mathcal{H}_{n} , \mathcal{E}_{n} ) \\ \hphantom{\mathcal{H}_{n+1} =}{}+ \frac{\tau \hbar ^{{\boldsymbol{\alpha }}}}{\Gamma ({\boldsymbol{\alpha }}+2)\mathsf{B}({\boldsymbol{\alpha }})} \sum_{\zeta =0}^{n} \bigl[ {t_{\zeta }^{\alpha -1}}\mathcal{F}_{2} (t_{\zeta },\mathcal{T}_{\zeta },\mathcal{H}_{\zeta }, \mathcal{E} _{ \zeta } ) \bigl[(-\zeta +1+n)^{{\boldsymbol{\alpha }}}(-\zeta +n+{ \boldsymbol{\alpha }}+2)\\ \hphantom{\mathcal{H}_{n+1} =}{}-(-\zeta +n)^{{\boldsymbol{\alpha }}}(-\zeta +n+2{\boldsymbol{\alpha }}+2) \bigr] \\ \hphantom{\mathcal{H}_{n+1} =}{}-{t_{\zeta -1}^{\alpha -1}}\mathcal{F}_{2} (t_{\zeta -1}, \mathcal{T}_{\zeta -1},\mathcal{H} _{\zeta -1}, \mathcal{E} _{\zeta -1} ) \bigl[(-\zeta +1+n)^{{\boldsymbol{\alpha }}+1} -(-\zeta +n)^{{\boldsymbol{\alpha }}}(-\zeta +n+ {\boldsymbol{\alpha }}+1) \bigr] \bigr], \end{gathered} \\& \begin{gathered} \mathcal{E}_{n+1} = \mathcal{E}_{0}+ \frac{\tau {t_{n}^{\alpha -1}}(1-{\boldsymbol{\alpha }})}{\Gamma ({\boldsymbol{\alpha }})} \mathcal{F}_{3} (t_{n }, \mathcal{T}_{n} , \mathcal{H}_{n} , \mathcal{E}_{n} ) \\ \hphantom{\mathcal{E}_{n+1} =}{}+\frac{\tau \hbar ^{{\boldsymbol{\alpha }}}}{\Gamma ({\boldsymbol{\alpha }}+2)\mathsf{B}({\boldsymbol{\alpha }})} \sum_{\zeta =0}^{n} \bigl[ {t_{\zeta }^{\alpha -1}}\mathcal{F}_{3} (t_{\zeta }, \mathcal{T}_{\zeta },\mathcal{H}_{\zeta }, \mathcal{E} _{ \zeta } ) \bigl[(-\zeta +1+n)^{{\boldsymbol{\alpha }}}(-\zeta +n+{\boldsymbol{ \alpha }}+2)\\ \hphantom{\mathcal{E}_{n+1} =}{}-(-\zeta +n)^{{\boldsymbol{\alpha }}}(-\zeta +n+2{\boldsymbol{\alpha }}+2) \bigr] \\ \hphantom{\mathcal{E}_{n+1} =}{}-{t_{\zeta -1}^{\alpha -1}}\mathcal{F}_{3} (t_{\zeta -1}, \mathcal{T}_{\zeta -1},\mathcal{H}_{\zeta -1}, \mathcal{E} _{\zeta -1} ) \bigl[(-\zeta +1+n)^{{\boldsymbol{\alpha }}+1} -(-\zeta +n)^{{\boldsymbol{\alpha }}}(-\zeta +n+ {\boldsymbol{\alpha }}+1) \bigr] \bigr], \end{gathered} \end{aligned}$$

where

80 $$ \begin{gathered} \mathcal{F}_{1} \bigl({\eta }, \mathcal{T}({\eta }),\mathcal{H}({\eta }), \mathcal{E}({\eta }) \bigr)= \mathcal{T}({\eta }) \bigl(1-\mathcal{T}({ \eta }) \bigr)-\beta _{12} \mathcal{T}({\eta })\mathcal{H}({\eta })- \beta _{13} \mathcal{T}({\eta })\mathcal{E}({\eta }), \\ \mathcal{F}_{2} \bigl({\eta },\mathcal{T}({\eta }),\mathcal{H}({\eta }), \mathcal{E}({\eta }) \bigr)= {\eta }_{1} \bigl[k_{2} \mathcal{H} ({ \eta }) \bigl(1-\mathcal{H}({\eta }) \bigr)-\beta _{21} \mathcal{T}({ \eta })\mathcal{H}({\eta }) \bigr] , \\ \mathcal{F}_{3} \bigl({\eta },\mathcal{T}({\eta }),\mathcal{H}({\eta }), \mathcal{E}({\eta }) \bigr)= \frac{k_{3} \mathcal{T}({\eta })\mathcal{E}({\eta })}{\mathcal{T}({\eta })+s_{3}}- \beta _{31} \mathcal{T}({\eta })\mathcal{E}({\eta })-c_{3} \mathcal{E}({ \eta }). \end{gathered} $$

### Numerical simulations

**Figure 16:**
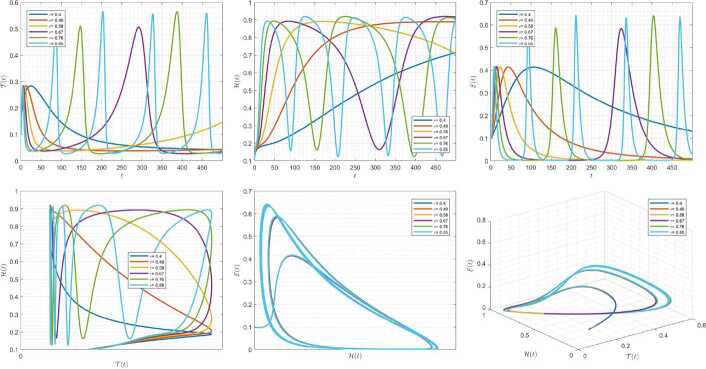
Simulations for solving (75) using (79) along with $(\mathcal{T}(0),\mathcal{H}(0),\mathcal{E}(0) )= (0.1,0.1,0.1 )$

**Figure 17:**
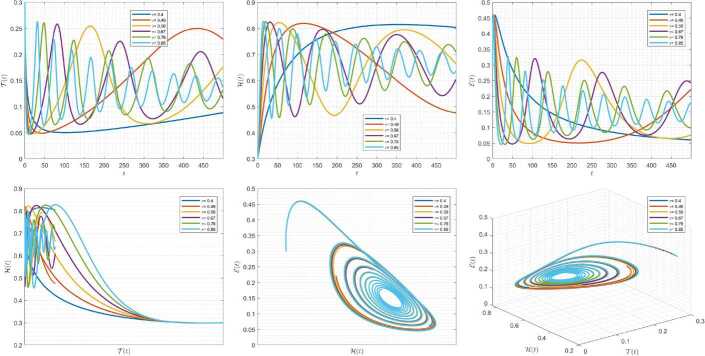
Simulations for solving (75) using (79) along with $(\mathcal{T}(0),\mathcal{H}(0),\mathcal{E}(0) )= (0.3,0.3,0.3 )$ and $\beta _{12}= 0.745$

**Figure 18:**
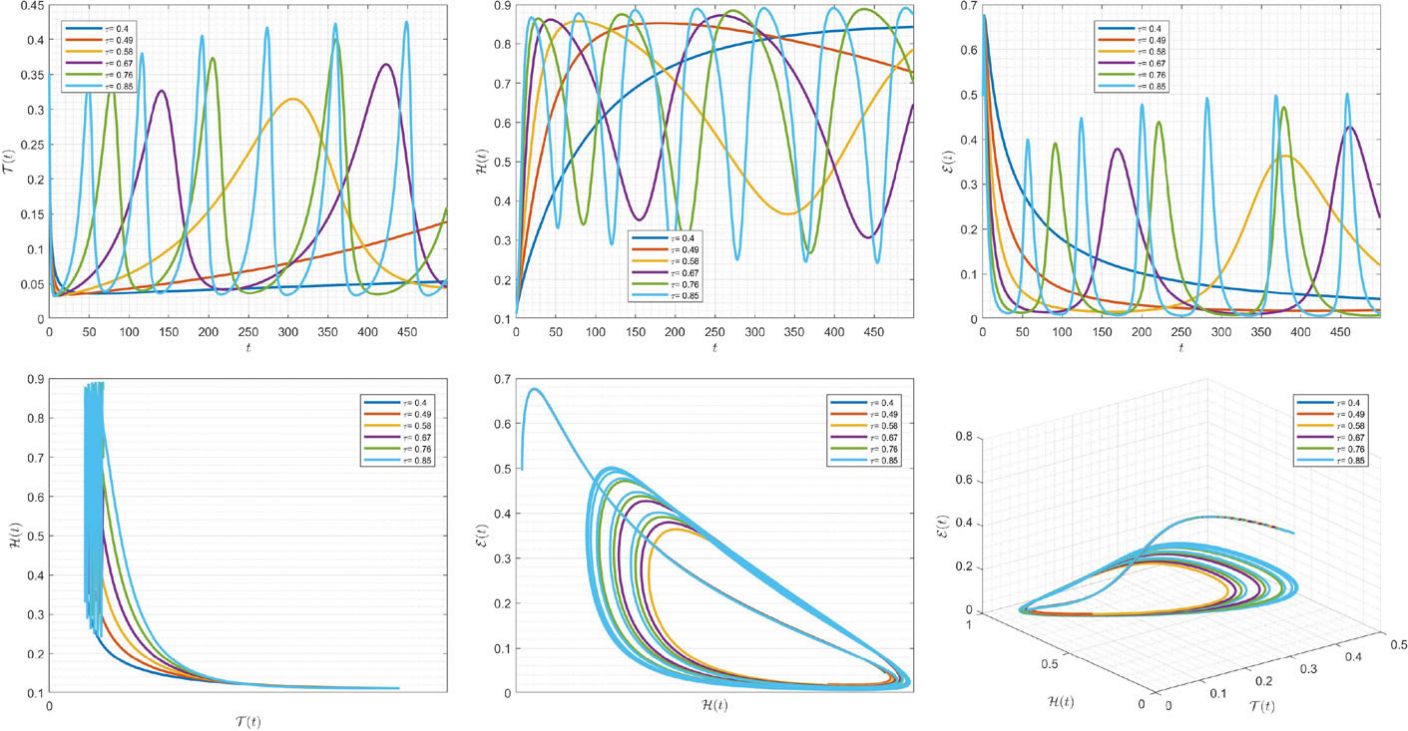
Simulations for solving (75) using (79) along with $(\mathcal{T}(0),\mathcal{H}(0),\mathcal{E}(0) )= ( 0.3517, 0.1115, 0.4951 )$ and $\beta _{12}= 0.92$

Figures 16–18 demonstrate the variation of state variables in model (75) when scheme (79) is utilized for different values of $\tau \in (0,1]$ and $\alpha =0.95$. In this simulations, we considered the following values in the model: $\beta _{12}=1$, $\beta _{13}=2.5$, $k_{2}=0.6$, $\beta _{21}=1.5$, $k_{3}=4.5$, $s_{3}=1$, $a_{31}=0.2$, and $d_{3}=0.5$. In our numerical simulations, $t_{\mathrm{final}}= 500$ and $\hbar = 0.001$. In Fig. 16, we take $(\mathcal{T}(t), \mathcal{H}(t), \mathcal{E}(t) )|_{t=0}= (0.1, 0.1, 0.1 )$. In this case the model exhibits chaotic attractor behavior. Also, by taking into consideration $(\mathcal{T}(t), \mathcal{H}(t), \mathcal{E}(t) )|_{t=0}= (0.3, 0.3, 0.3 )$ and $\beta _{12} = 0.745 $ the model shows the limit cycle behavior in Fig. 17, whereast for $\mathcal{T}(0)=0.3517$, $\mathcal{H}(0)=0.1115$, $\mathcal{E}(0)=0.4951$, and $\beta _{12} = 0.920$, we get periodic orbit trajectories in the solutions as depicted in Fig. 18.

**Figure 19:**
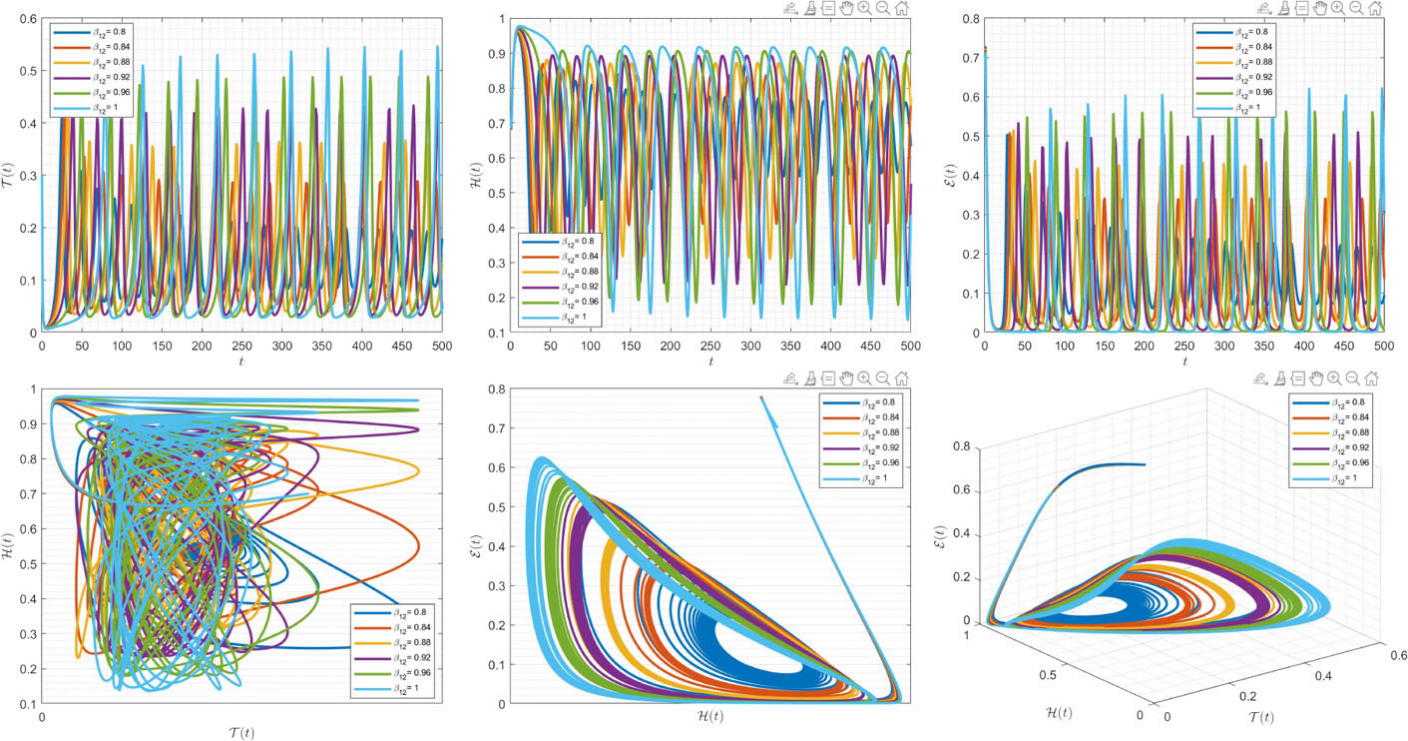
Simulations for solving (43) using (59) along with $(\mathcal{T}(0),\mathcal{H}(0),\mathcal{E}(0) )= (0.3, 0.7, 0.7 )$ and $\alpha =0.98 $ for different $\beta _{12}$

**Figure 20:**
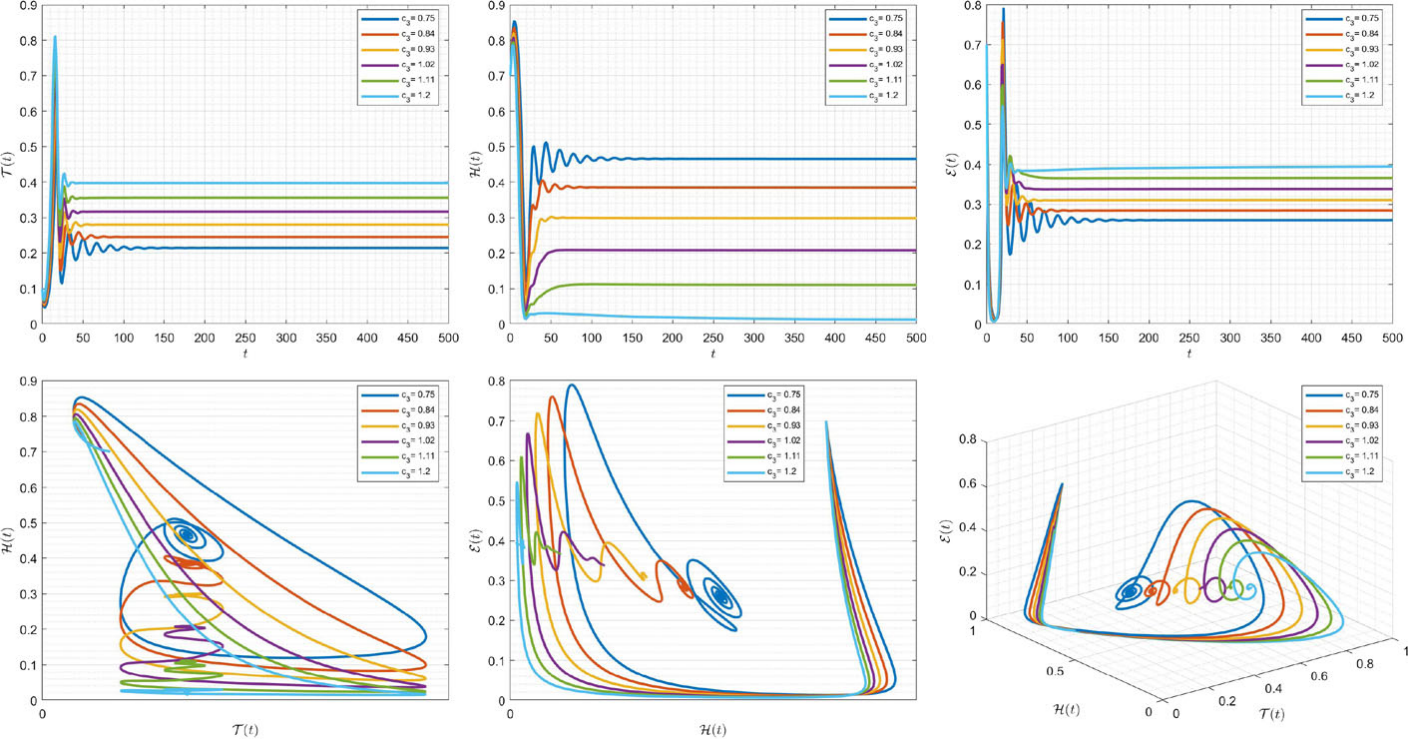
Simulations for solving (43) using (59) along with $(\mathcal{T}(0),\mathcal{H}(0),\mathcal{E}(0) )= (0.3, 0.7, 0.7 )$ and $\alpha =0.98 $ for different $c_{3}$

In Figs. 19 and 20, we also investigate the sensitivity of the state variables when two parameters $\beta _{12}$ and $c_{3}$ change, respectively. In these two figures, we can see that changes in each of these parameters cause changes that are somewhat meaningful and constructive in the behavior of variables. As a biological conclusion, we can point to the fact that the stability in the model can only be achieved when the recruitment rate of effector cells is greater than the rate of inactivation by cancer cells. In other words, if the immune system is unable to detect and attack cancer cells, then effective treatment must be used to control the tumor growth.

## Conclusion

Recent advances in the presentation of efficient numerical methods in fractional differential calculus are a great help to researchers in modeling real phenomena. It has also been proven that differential calculus of integer order in some cases is incapable of describing the behavior of some phenomena or faces fundamental problems. In this paper, we study the dynamics of a tumor-immune model due to the unpredictable growth of tumor cells described by an attractive form of nonlinear differential equations. The importance of this model has led to many different approaches of studying the problem. Our study in the paper makes it clear how tumor cells interact with the immune system. The main difference of this paper from other papers on this system is that we have used new fractional and fractal-fractional definitions for the derivative in the system structure. Some of these new concepts of differentiation were introduced in 2017 by Atangana and his collaborators. They combine the idea of fractal derivative and fractional differentiation, which takes into account the memory, fractal effect, and nonlocality. The model considers processes like power-law, fading memory, and crossover. It is important to note that the numerical methods and analysis used in this paper are quite different from those presented in [24]. In fact, this work can be considered as a complement to the content presented in this paper. The basis of these mathematical algorithms is applying some fundamental axioms of fractional derivative and the first-order interpolation. The existence and uniqueness of the model solutions were also investigated. The interesting attractors obtained in this paper imply that these new fractional and fractal-fractional operators can describe newer aspects of the behavior of these systems than fractional derivatives. Some of these features cannot be described by conventional integer-order operators. Through numerical simulations, we have confirmed the chaotic dynamics of the model by taking certain parameters in the model and suitable initial conditions. These results indicate that the values considered for the fractional-order derivatives significantly influence the dynamic behavior of the problem. The fractal-fractional operators allow us to describe self-similar problems with power-law, fading memory, and crossover behavior. In addition, the chaotic behavior seen in the results is entirely consistent with the inherent nature of the problem. These systems are recognized as complex real-world problems that cannot be represented with classical or fractional differential operators. Numerical simulations confirm that a change in fractional order exhibits memory properties and very strange complex dynamical behaviors. The results of this paper show that other similar problems in this field can be described and investigated using the numerical methods used in this paper.

## Data Availability

Not applicable.
